# Salicylic acid receptors activate jasmonic acid signalling through a non-canonical pathway to promote effector-triggered immunity

**DOI:** 10.1038/ncomms13099

**Published:** 2016-10-11

**Authors:** Lijing Liu, Fathi-Mohamed Sonbol, Bethany Huot, Yangnan Gu, John Withers, Musoki Mwimba, Jian Yao, Sheng Yang He, Xinnian Dong

**Affiliations:** 1 Howard Hughes Medical Institute-Gordon and Betty Moore Foundation, Department of Biology, Duke University, Durham, North Carolina 27708, USA; 2 Department of Basic Sciences, Faculty of Dentistry, Sinai University, Al Arish, North Sinai 45518, Egypt; 3 Department of Energy Plant Research Laboratory, and Cell and Molecular Biology Program, Michigan State University, East Lansing, Michigan 48824, USA; 4 Howard Hughes Medical Institute, Department of Energy Plant Research Laboratory, and Department of Plant Biology, Michigan State University, East Lansing, Michigan 48824, USA; 5 Department of Biological Sciences, Western Michigan University, Kalamazoo, Michigan 49008, USA

## Abstract

It is an apparent conundrum how plants evolved effector-triggered immunity (ETI), involving programmed cell death (PCD), as a major defence mechanism against biotrophic pathogens, because ETI-associated PCD could leave them vulnerable to necrotrophic pathogens that thrive on dead host cells. Interestingly, during ETI, the normally antagonistic defence hormones, salicylic acid (SA) and jasmonic acid (JA) associated with defence against biotrophs and necrotrophs respectively, both accumulate to high levels. In this study, we made the surprising finding that JA is a positive regulator of RPS2-mediated ETI. Early induction of JA-responsive genes and *de novo* JA synthesis following SA accumulation is activated through the SA receptors NPR3 and NPR4, instead of the JA receptor COI1. We provide evidence that NPR3 and NPR4 may mediate this effect by promoting degradation of the JA transcriptional repressor JAZs. This unique interplay between SA and JA offers a possible explanation of how plants can mount defence against a biotrophic pathogen without becoming vulnerable to necrotrophic pathogens.

## Abstract (web summary)

Salicylic acid (SA) and jasmonic acid (JA) often act antagonistically in plant defence. Here, Liu *et al*. show that during effector-triggered immunity (ETI) against *Pseudomonas syringae*, JA signalling is activated via a non-canonical pathway involving the SA receptors, NPR3 and NPR4, to positively regulate ETI.

## Graphical abstract

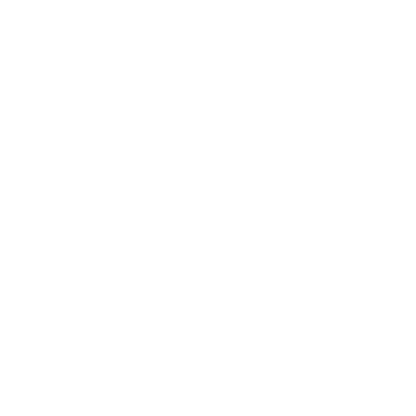

Plants are constantly exposed to a wide range of microbial pathogens and herbivorous insects with different and sometimes opposing infection mechanisms. Therefore, they have to adjust their defence strategies accordingly through complex interplay between different phytohormones. Of those, salicylic acid (SA) is a major defence hormone against biotrophic and hemibiotrophic pathogens, which rely on living plant tissue for nutrients^1^,^2^,^3^. In contrast, plants produce jasmonic acid (JA) in response to wounding caused by insects and to necrotrophic microbes which obtain nutrients from dead host cells^3^,^4^,^5^. The crosstalk between SA and JA was first observed in tomato where SA- or acetyl-SA treatment blocked not only JA production but also JA-mediated transcription of protease inhibitors involved in defence against insects^6^,^7^. Co-treating *Arabidopsis* with SA and JA abolished JA-mediated induction of the defensin gene *PDF1.2* (refs 8, 9), while infection by the hemibiotrophic bacterial pathogen *Pseudomonas syringae.* pv. *tomato* (*Pst)* DC3000, which enhanced SA production, led to reduced resistance to the necrotrophic fungal pathogen *Alternaria brassicicola* in the neighbouring tissue^10^. Conversely, mutants deficient in JA signalling were found to be more resistant to *Pst* DC3000 (refs 11, 12). These studies clearly support the existence of SA–JA antagonism in basal resistance as a mechanism to prioritize the defence strategy according to the type of pathogen encountered. At the molecular level, this antagonism depends on the SA-signalling component nonexpressor of PATHOGENESIS-RELATED GENE 1 (*NPR1*) and the downstream transcription factors TGAs and WRKYs^9^,^13^,^14^,^15^; and the JA-inducible NAC transcription factors regulating SA biosynthesis and metabolism^16^.

Despite this well-documented reciprocal inhibition, the relationship between SA and JA is not always antagonistic. Studies in rice demonstrated that JA signalling positively regulates plant resistance to the biotrophic pathogen, *Xanthomonas oryzae* pv. *oryzae* (*Xoo*)^17^, possibly due to a common defence system activated by both SA and JA^18^. Mur *et al*. found that the outcome of the SA–JA interaction depends on relative concentrations of the two hormones^19^. Moreover, the antagonism between *Pst* DC3000 and *A. brassicicola* was not observed if *Pst* DC3000 carried the *avrRpt2* effector gene^10^, which causes effector-triggered immunity (ETI) in the infected tissues. During this ETI induction, a high level of SA was produced, but no crosstalk suppression on JA signalling was detected^10^.

ETI is a major innate immune mechanism in plants against biotrophic pathogens that occurs when the activity of a pathogen effector delivered into the host cell is detected by a host nucleotide-binding leucine-rich repeat (NB-LRR) immune receptor^20^,^21^. ETI triggers a dramatic immune response that often leads to sacrifice of the infected cells in the form of programmed cell death (PCD). In parallel to SA accumulation, the endogenous JA level also increases during ETI induction^19^,^22^, which may explain why ETI-associated PCD does not enhance plant susceptibility to necrotrophic pathogens in the neighbouring tissue^10^. Understanding the cooperative interplay between SA and JA during ETI is, therefore, essential for elucidating the molecular mechanism of this key immune response in plants.

While the role of JA in ETI remains to be investigated, the molecular function of SA in ETI has been illustrated by the discovery of SA receptors, NPR3 and NPR4 (NPR3/4), which are substrate adaptors for Cullin 3 (Cul3) ubiquitin E3 ligases. The high concentration of SA generated during ETI has been shown to promote the degradation of NPR1 (ref. 23). Even though NPR1 is a positive regulator of SA-mediated basal resistance, it is a repressor of ETI^23^,^24^. During ETI, degradation of NPR1 is mediated by NPR3 whose interaction with NPR1 depends on high levels of SA^23^. A failure to remove NPR1 repression in *npr3* explains the ETI deficiency observed in this mutant. However, NPR1 may not be the only repressor that needs to be removed for ETI activation because the mutant of another SA receptor, NPR4, which does not interact with NPR1 at high levels of SA, was similarly deficient in ETI, and the *npr3 npr4* double mutant had a more severe ETI deficiency than the single mutants^23^. Moreover, mutating NPR1 in *npr3 npr4* could not fully restore ETI and PCD in the *npr1 npr3 npr4* triple mutant, suggesting that NPR3 and NPR4 have additional substrates, besides NPR1, that are involved in this major immune response.

Similar to the SA receptors NPR3 and NPR4, which are adaptors for the Cul3 E3 ubiquitin ligase, the canonical JA receptor complex contains COI1 (CORONATINE INSENSITIVE 1), an F-box protein for the Cul1 E3 ubiquitin ligase, and one of the 12 JAZ (JASMONATE ZIM DOMAIN) proteins^25^. Interaction between COI1 and JAZs requires the presence of the biologically active (+)-7-*iso*-jasmonoyl-L-isoleucine (JA-Ile)^26^. On perception of JA-Ile, JAZ proteins are ubiquitinated by SCF^COI1^ and degraded by the 26S proteasome^11^,^27^ to release their repression on transcription factors, such as MYCs, which are activators of JA-responsive genes^28^,^29^. Although the *coi1-1* mutant has been reported to display enhanced resistance to *Pst* DC3000/*avrRpm1*, it was hypothesized to be due to the high basal defence in this mutant^30^. Thus, whether and how these JA signalling components are involved in the ETI-specific response needs to be explored.

In this study, we show that JA signalling positively regulates the establishment of RPS2-mediated ETI. However, the initial activation of JA-responsive genes during ETI is dependent on SA and SA receptors NPR3 and NPR4, but not on NPR1 or the canonical JA receptor COI1. ETI-mediated *de novo* JA synthesis is also compromised in an *npr3 npr4* mutant. We further showed that NPR3 and NPR4 can interact with JAZ proteins in a manner that is enhanced by SA, and promote the reduction of JAZ1 protein levels at the early stage of ETI. This unique mechanism of reducing JAZ levels via NPR3/NPR4 provides an explanation for the observations above. Our study, therefore, reveals a new and intricate interplay between the two major plant defence hormone pathways.

## Results

### JA synthesis and signalling positively regulate ETI

**Figure 1:**
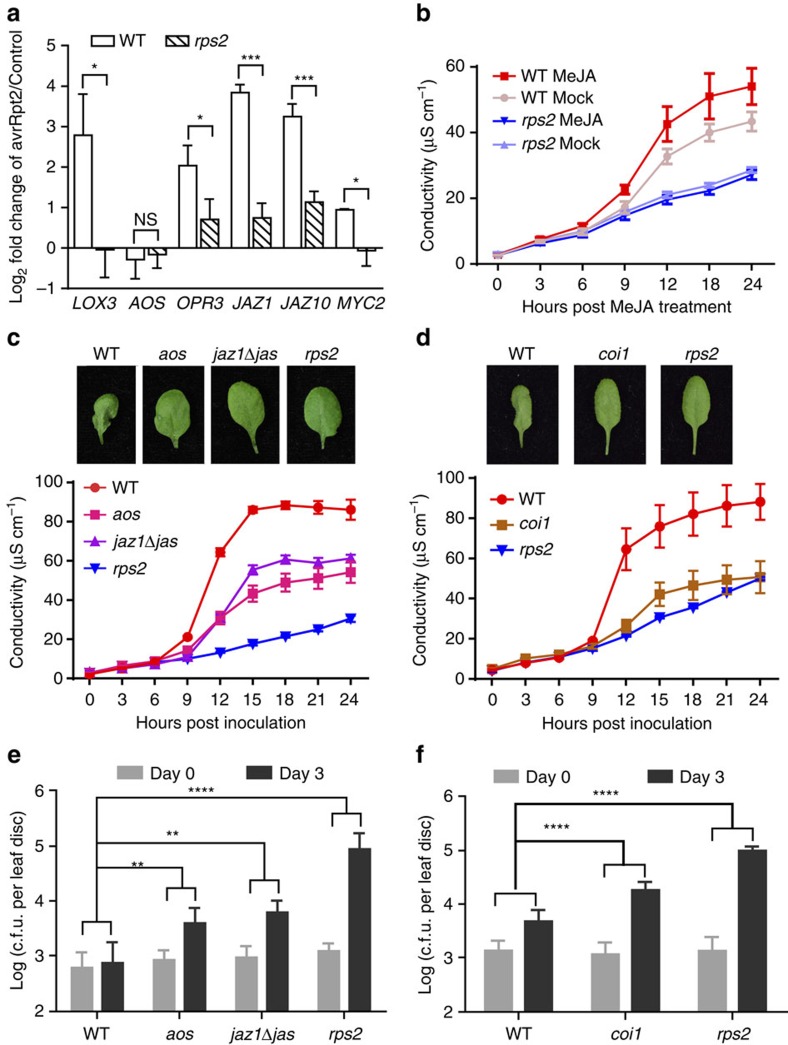
JA synthesis and signalling are activated through the RPS2 immune receptor and required for ETI and associated PCD. (**a**) Expression levels of JA-responsive genes in wild-type (WT) and the *rps2* mutant at 4 h post inoculation (h.p.i.) with *Psm* ES4326/*avrRpt2* at OD_600nm_=0.2. The final data were normalized to the expression with 10 mM MgSO_4_ (Control) treatment. qRT-PCR was performed on *LOX3* (*LIPOXYGENASE 3*), *AOS* (*ALLENE OXIDE SYNTHASE*), *OPR3* (*OPDA REDUCTASE 3*), *JAZ1* (*JASMONATE ZIM-DOMAIN PROTEIN 1*), *JAZ10*, *MYC2* (*ARABIDOPSIS MYELOCYTOMATOSIS ONCOGENE HOMOLOG 2*) with *UBQ5* (*UBIQUITIN 5*) as a reference. Data from three biological replicates were combined using linear mixed-effects model. Significant difference was detected using Student's *t*-test. Data are presented as Mean±s.d. (**b**) Three-week-old plants were first infiltrated with *Psm* ES4326/*avrRpt2* at OD_600nm_=0.01, and 3.5 h later water (Mock) or 100 μM MeJA (MeJA) was sprayed. Starting at 0.5 h post inoculation (h.p.i.), leaf discs were collected for conductivity assay. Data are shown as mean±s.d. (*n*=3 biological replicates). (**c**) Representative leaves of WT, *aos*, *jaz1Δjas*, and *rps2* plants 15 h.p.i. by *Psm* ES4326/*avrRpt2* at OD_600nm_=0.01 (upper panel). Conductivity measurements were performed 0.5 h.p.i. with *Psm* ES4326/*avrRpt2* (lower panel). Data are shown as mean±s.d. (*n*=3 biological replicates). (**d**) Representative leaves and conductivity measurements of WT, *coi1* and *rps2* plants. The pathogen inoculation and conductivity measurements were as described in **c**. Data are shown as mean±s.d. (*n*=3 biological replicates). (**e**) Plants were infiltrated with *Psm* ES4326/*avrRpt2* at OD_600nm_=0.002 and pathogen growth was measured at day 0 and day 3. c.f.u., colony forming unit. Significant difference was detected by two-way ANOVA. Data are shown as mean±s.d. (*n*=8 biological replicates). (**f**) The growth of *Psm* ES4326/*avrRpt2* in WT, *coi1*, and *rps2*. The same method was used as described in **e**. All experiments were repeated three times with similar results. *P<0.05; **P<0.01; ***P<0.001; ****P<0.0001; NS, no significant difference. qRT-PCR, quantitative reverse transcription PCR.

To test our hypothesis that the JA signalling pathway is an additional level of regulation during ETI, we first examined the expression of several primary JA-responsive genes^31^ at 4 h post inoculation (h.p.i.) by *Ps* pv. *maculicola* (*Psm*) ES4326 carrying the effector *avrRpt2* (ref. 32). As shown in Fig. 1a, five of the six genes tested were induced by *Psm* ES4326/*avrRpt2* in wild-type (WT) *Arabidopsis* plants. In comparison, the induction of these genes was drastically compromised in the immune receptor mutant, *rps2,* which cannot recognize the activity of avrRpt2 (ref. 32), while the basal expression levels of these genes in WT and *rps2* were comparable (Supplementary Fig. 1). This dependence on immune receptor is not specific to RPS2, a CC-NB-LRR receptor^33^; a similar effect was also observed for the TIR-NB-LRR immune receptor RPS4 based on analysis of a previously published microarray data set^34^ (Supplementary Fig. 2). JA-responsive genes were induced in *35S:RPS4-HS* in the WT background after treatment, while in the *eds1* mutant background compromised in RPS4-mediated ETI, the activation of these genes was blocked.

To further reveal the function of JA signalling during ETI, we sprayed plants with Methyl-JA (MeJA) 3.5 h after Psm ES4326/*avrRpt2* infiltration and monitored ETI-associated PCD by measuring ion leakage (an indicator of cell death) during a 24-h time course. We observed that MeJA could further accelerate PCD in WT plants, but not in the *rps2* mutant (Fig. 1b). Next, we measured ion leakage in three JA mutants: *aos* (*ALLENE OXIDE SYNTHASE*), a JA-deficient mutant^35^,^36^; *coi1*, the JA receptor mutant^25^; and *jaz1Δjas,* a transgenic line over-expressing a JAZ1 protein that lacks the Jas domain and exhibits a constitutive repressor activity on JA signalling^11^. Homozygous *coi1* plants were selected on MS media supplemented with JA (Supplementary Fig. 3). These mutants all showed compromised PCD compared to WT on *Psm* ES4326*/avrRpt2* challenge (Fig. 1c, d), consistent with a positive role of JA in ETI. In addition, resistance triggered by the *avrRpt2* effector was also diminished in *aos*, *coi1* and *jaz1Δjas* in comparison to the WT control (Fig. 1e,f). Collectively, these data demonstrate that JA synthesis and signalling are positive regulators of RPS2-mediated ETI.

**Figure 2:**
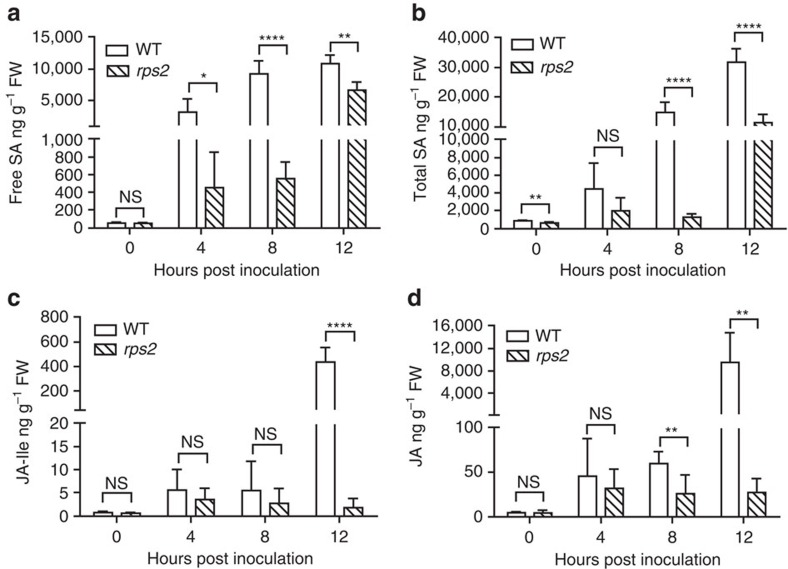
The levels of SA and JA in WT and *rps2* during ETI. Three-week-old WT and *rps2* plants were infiltrated with *Psm* ES4326*/avrRpt2* at OD_600nm_=0.01. Samples were collected at 0, 4, 8, 12 h.p.i. The levels of (**a**) free SA; (**b**) total SA; (**c**) JA-Ile; and (**d**) JA were measured. Significant difference was detected using Student's *t*-test. Data are shown as mean±s.d. (*n*=5–6 biological replicates). All experiments were repeated twice with similar results. *P<0.05; **P<0.01; ****P<0.0001; NS, no significant difference.

According to our results in Fig. 1, we made the assumption that during ETI, *de novo* JA synthesis occurs before effector-triggered PCD which is normally observed after 12 h.p.i. To test this hypothesis, we measured the concentrations of free SA, SA O-β-glucoside (SAG), JA-Ile and JA at 0, 4, 8, and 12 h after infiltrating WT and *rps2* with *Psm* ES4326*/avrRpt2*. As shown in Fig. 2, in response to pathogen inoculation, there were a sequential increase in these measured hormones with free SA starting as early as 4 h.p.i. (Fig. 2a), total SA (SA+SAG) and JA at 8 h.p.i. (Fig. 2b,d), and then JA-Ile at 12 h.p.i. (Fig. 2c). These increases were significantly dampened in the *rps2* mutant, indicating that they were ETI-specific. We also observed an ETI-induced ABA accumulation initiating at 4 h.p.i. (Supplementary Fig. 4).

### Early JA induction in ETI is principally dependent on NPR3/4

**Figure 3:**
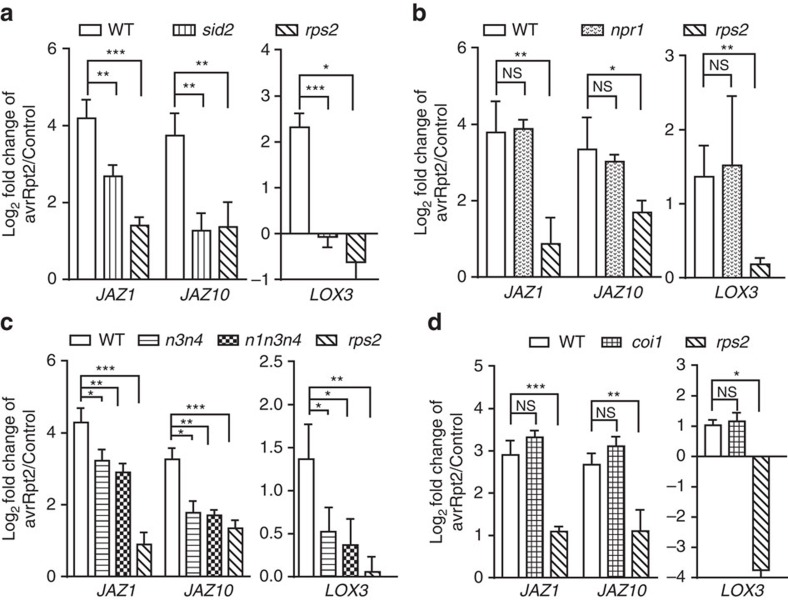
ETI-mediated early induction of JA-responsive genes is dependent on SA and NPR3 and NPR4 but independent of NPR1 and the JA receptor COI1. Leaves from corresponding plants were harvested 4 h.p.i. with *Psm* ES4236/*avrRpt2* (avrRpt2) at OD_600nm_=0.2 or 10 mM MgSO_4_ (Control). qRT-PCR was performed on *LOX3*, *JAZ1*, *JAZ10*, with *UBQ5* as a reference. Gene expression (**a**) in WT, *sid2* and *rps2*; (**b**) in WT, *npr1* and *rps2*; (**c**) in WT, *npr3 npr4* (*n3n4*), *npr1 npr3 npr4* (*n1n3n4*) and *rps2*; (**d**) in WT, *coi1* and *rps2* was measured. Data from three biological replicates were combined using linear mixed-effects model. Significant difference was detected using Student's *t*-test. Data are shown as mean±s.d. *P<0.05; **P<0.01; ***P<0.001; NS, no significant difference. qRT-PCR, quantitative reverse transcription PCR.

Since SA and JA are sequentially induced during ETI, we next examined a possible connection between these two hormones by analysing the expression of JA-responsive genes 4 h.p.i. by *Psm* ES4326/*avrRpt2* in the SA-deficient *sid2* mutant^2^. Contrary to the expected antagonism of SA on the JA response, the induction of *JAZ1*, *JAZ10* and *LOX3* was inhibited in the *sid2* mutant similar to the level observed in the *rps2* mutant (Fig. 3a). These data showed that SA is a necessary signal for this early induction of JA signalling during RPS2-mediated ETI. As NPR1 has been proposed as an SA receptor^37^,^38^, and a major regulator of both SA signalling^1^ and SA-mediated repression of the JA response^9^, we next tested whether NPR1 is also involved in this SA-dependent JA signalling activation at the early stage of ETI. Our result showed that mutating NPR1 had little effect on the ETI-triggered induction of these JA-responsive genes (Fig. 3b).

On the basis of the report that NPR3 and NPR4 are the SA receptors functionally required for ETI^23^, we analysed the expression of JA-responsive genes in the *npr3 npr4* double mutant. We found that in the *npr3 npr4* mutant, the early induction of *JAZ1*, *JAZ10* and *LOX3* at 4 h.p.i. was compromised (Fig. 3c). This result indicates that the activation of JA signalling at the early stage of RPS2-mediated ETI principally depends on NPR3/4, but is independent of NPR1. Consistent with this conclusion, comparable expression of JA-responsive genes was detected in *npr3 npr4* and *npr1 npr3 npr4* (Fig. 3c). The basal expression of these JA-responsive genes and the SA-mediated repression of JA-induced *PDF1.2* expression were intact in the *npr3 npr4* mutant (Supplementary Figs 5 and 6), indicating that NPR3/4 affect JA signalling only during ETI.

Activation of the canonical JA signalling pathway involves degradation of JAZs through the JA receptor COI1, an F-box protein of an SCF-type E3 ubiquitin ligase^11^,^39^. To test if ETI-mediated early induction of JA signalling is dependent on *de novo* JA synthesis and canonical JA signalling, we checked the expression of JA-responsive genes in the *aos, coi1* and *jaz1Δjas* mutants. Surprisingly, the induction of JA-responsive genes at 4 h.p.i. by *Psm* ES4326/*avrRpt2* was abolished only in *jaz1Δjas,* but not in *aos* or *coi1* (Fig. 3d; Supplementary Fig. 7). On the contrary, the induction of these genes was even higher in *aos* than in WT, likely due to the higher level of SA in JA mutant^12^. These results indicate that the ETI-mediated early induction of JA signalling is independent of *de novo* JA synthesis, but dependent on the degradation of JAZs, and occurs through a non-canonical pathway in which NPR3 and NPR4, instead of COI1, play an essential role.

### Interactions between NPR3/4 and JAZs are enhanced by SA

**Figure 4:**
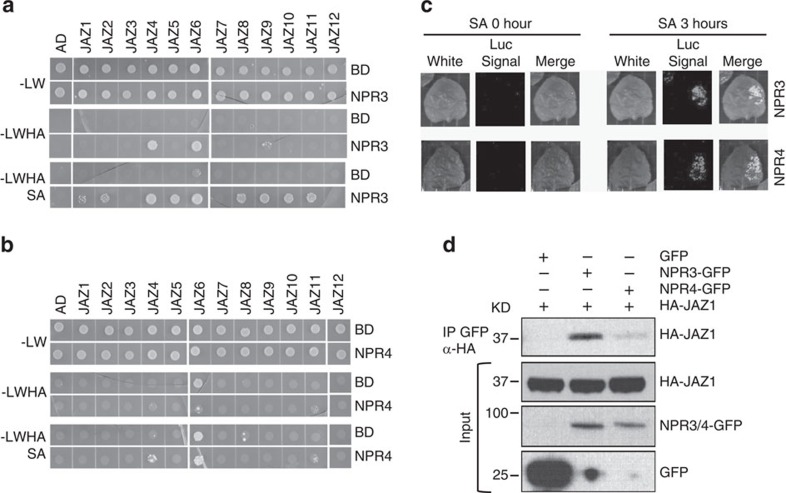
SA facilitates NPR3 and NPR4 interactions with JAZ proteins. Yeast two-hybrid assay (Y2H) was performed to study (**a**) interactions between NPR3 and JAZs and (**b**) interactions between NPR4 and JAZs. For SA treatment, 100 μM SA was added in the yeast media. (**c**) The split-luciferase assays were performed on JAZ1 with NPR3 or NPR4. The left half of the *N. benthamiana* leaf was co-infiltrated with *cLuc* and *NPR3-nLuc* or *cLuc* and *NPR4-nLuc* as control. The right half of the leaf was co-infiltrated with *cLuc-JAZ1* and *NPR3-nLuc* or *cLuc-JAZ1* and *NPR4-nLuc*. After two days of incubation, luciferin was sprayed onto the inoculated leaves and chemiluminescence images were taken by a CCD camera 0 and 3 h after 1 mM SA treatment. (**d**) The co-immunoprecipitation (co-IP) assay on JAZ1 with NPR3 or NPR4 in *Arabidopsis*. Samples were collected at 4 h.p.i. with *Psm* ES4326/*avrRpt2,* at OD_600nm_=0.2, plus 40 μM MG115. The co-IP assay was carried out using the GFP-Trap_A beads for 2 h at 4 °C. The HA-JAZ1 protein was detected by western blot using HA antibody. The NPR3-GFP, NPR4-GFP or GFP protein levels were measured by western blots using the GFP antibody. All experiments were repeated three times with similar results.

JAZ proteins are the central transcriptional repressors of JA signalling^27^,^40^. On the basis of our discovery that NPR3/4 are required for early activation of JA signalling during RPS2-mediated ETI, we hypothesized that these proteins might serve as adaptors for an E3 ubiquitin ligase that targets the JAZ proteins for degradation. To test our hypothesis, we first performed yeast two-hybrid (Y2H) assays. Each of the twelve JAZ proteins was fused with the transcriptional activation domain (AD), and NPR3 and NPR4 were fused with the DNA binding domain (BD). As shown in Fig. 4a, nine of the 12 JAZ proteins interacted with NPR3. Six of these nine interactions required the presence of SA (JAZ1, 2, 5, 8, 10, 11), two were enhanced by SA (JAZ4 and JAZ9) and one was SA independent (JAZ6). In addition to NPR3, two JAZ proteins (JAZ4 and JAZ11) were also found to interact with NPR4 (Fig. 4b).

We next used a split luciferase assay to verify these interactions *in planta*. JAZ1 and NPR3 were fused with C-terminal (cLuc) and N-terminal (nLuc) halves of luciferase, respectively, to generate cLuc-JAZ1 and NPR3-nLuc, and were transiently co-expressed in leaves of *Nicotiana benthamiana*. The complemented luciferase activity was detected with the combination of cLuc-JAZ1 and NPR3-nLuc after SA treatment (Fig. 4c upper panel; Supplementary Fig. 8a). Even though NPR4 could not interact with JAZ1 in yeast, it showed a similar interaction pattern with JAZ1 in the split luciferase assay (Fig. 4c lower panel; Supplementary Fig. 8b). The same assay was applied to JAZ4, 6, 8 and 9 and all of them showed SA-enhanced interactions with both NPR3 and NPR4 (Supplementary Fig. 8). The SA-dependency for most of the NPR4-JAZ interactions is surprising because unlike NPR3, NPR4 interaction with NPR1 is constitutive until disrupted by SA^23^. However, this result is consistent with our hypothesis that both NPR3 and NPR4 can serve as ubiquitin E3 ligase adaptors for substrates other than NPR1.

To further confirm NPR3/4-JAZ1 interactions, we performed coimmunoprecipitation (co-IP) and pull down assays. We found that when JAZ1 was co-expressed with GFP, NPR3-GFP or NPR4-GFP in *N. benthamiana* leaves or in *Arabidopsis* transgenic lines, it was able to co-IP with NPR3 and NPR4 (Fig. 4d; Supplementary Fig. 9). To study the effect of SA on these interactions, we expressed NPR3-GFP and NPR4-GFP proteins in the *N. benthamiana* transgenic line carrying the salicylate hydroxylase gene, *NahG*. As shown in Supplementary Fig. 10, SA, but not JA, could enhance the pulldown of NPR3-GFP and NPR4-GFP by the purified recombinant GST-JAZ1. Interestingly, the NPR3-GFP and NPR4-GFP pulled down by GST-JAZ1 had higher-molecular weights than expected (Supplementary Fig. 10), probably due to unknown modifications. Collectively these data demonstrated that NPR3 and NPR4 could bind to JAZ proteins and SA could enhance the binding.

To determine which domains of JAZ1 and NPR3 are responsible for their interaction, we made truncated versions of both proteins. For JAZ1, we generated individual deletions of each of the three protein interacting domains, namely the N-terminal domain (NT), the zinc-finger expressed in inflorescence meristem domain (ZIM) and the jasmonate-associated domain (Jas)^41^. For NPR3, we separately removed the Bric-a-Brac, Tramtrack, Broad-complex (BTB) domain and the ankyrin repeat (ANK) domain, known to be involved in protein-protein interaction^23^, and the N- and C-terminal regions (Supplementary Fig. 11a). These JAZ1 and NPR3 constructs were then fused with the AD and BD domain, respectively, for Y2H experiments. We found that even though all of the mutant proteins expressed well in yeast (Supplementary Fig. 11b), deleting any of the domains in JAZ1 or NPR3 compromised their interaction, except deletion of the N-terminal region of NPR3, which made the interaction constitutive (Supplementary Fig. 11c). These data showed that the overall protein integrity for both JAZ1 and NPR3 is important for their interaction while the N-terminal region of NPR3 may impose an inhibitory effect.

### NPR3/4 promote the reduction of JAZ1 levels during ETI

**Figure 5:**
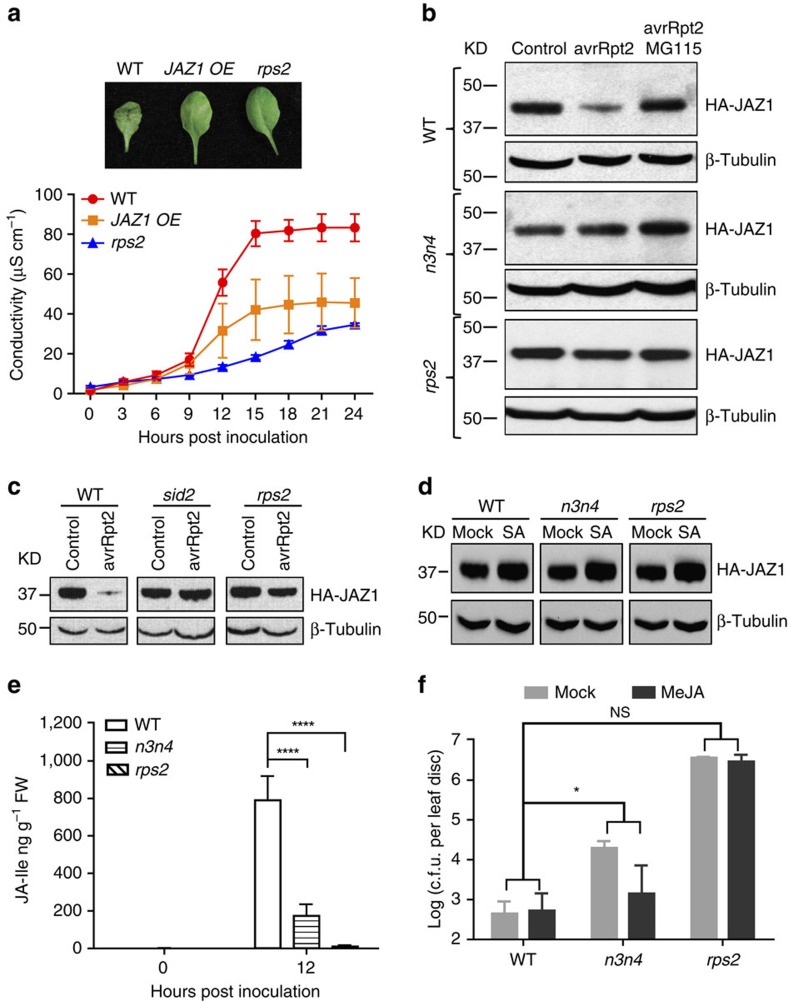
NPR3 and NPR4 promote ETI-induced reduction of JAZ1 level and *de novo* JA synthesis. (**a**) Representative leaves and conductivity measurements of WT, the *35S:HA-JAZ1* overexpressing (*JAZ1 OE*) transgenic line, and the *rps2* mutant. The same methods were used as in Fig. 1c. Data are shown as mean±s.d. (*n*=3 biological replicates). (**b**) HA-JAZ1 protein levels were determined in WT, *npr3 npr4* (*n3n4*) and the *rps2* mutant 4 h.p.i. with 10 mM MgSO_4_ (Control), *Psm* ES4326/*avrRpt2* (avrRpt2) or *Psm* ES4326/*avrRpt2* together with 40 μM MG115 (avrRpt2 MG115). (**c**) HA-JAZ1 protein levels were measured in WT, *sid2* and the *rps2* mutant 4 h.p.i. with 10 mM MgSO_4_ (Control), or *Psm* ES4326/*avrRpt2* (avrRpt2). (**d**) HA-JAZ1 protein levels were determined in WT, *npr3 npr4* (*n3n4*) and the *rps2* mutant 4 h after being sprayed with water (Mock) or 1 mM SA. For (**b**,**c**,**d**) the western blots were performed using the HA antibody. β-Tubulin served as a loading control. KD, kilodalton. (**e**) The JA-Ile levels in WT, *npr3 npr4* and *rps2* at 0 and 12 h.p.i. with *Psm* ES4326/*avrRpt2* at OD_600nm_=0.01. Significant difference was detected using Student's *t*-test. Data are shown as mean±s.d. (*n*=5–6 biological replicates). (**f**) Plants were infiltrated with *Psm* ES4326/*avrRpt2* at OD_600nm_=0.01 and 3.5 h later water (Mock) or 100 μM MeJA (MeJA) was sprayed. Pathogen growth was measured at 1 day post inoculation. Significant difference was detected by two-way ANOVA. Data are shown as mean±s.d. (*n*=8 biological replicates). All experiments were repeated three times with similar results. *P<0.05; ****P<0.0001; NS, no significant difference.

The deficiency in RPS2-mediated ETI observed in the *jaz1Δjas* transgenic line (Fig. 1c,e) might be due to stabilization of the mutant JAZ1 repressor in the absence of the Jas domain, which is required for interaction with its canonical partner, COI1 (ref. 39). Alternatively, the phenotype might result from a lack of interaction with NPR3/4, which might also affect JAZ1 stability, albeit through a different E3 ubiquitin ligase complex (that is, Cullin 3 instead of Cullin 1). To test which hypothesis is true, we first demonstrated that JAZ1 is a repressor of ETI by over-expressing (OE) HA-tagged JAZ1. We found that in response to *Psm* ES4326/*avrRpt2* inoculation, the *35S:HA-JAZ1* OE plants were impaired in effector-triggered PCD (Fig. 5a) similar to the *jaz1Δjas* transgenic line (Fig. 1c).

On the basis of our findings that both NPR3 and NPR4 interact with JAZ proteins in an SA-enhanced manner and the *npr3 npr4* double mutant has a more severe ETI deficiency than the *npr3* and *npr4* single mutants, we proposed that NPR3 and NPR4 function redundantly to degrade JAZ proteins during ETI^23^. To test this, we crossed the *35S:HA-JAZ1* OE line into the *npr3 npr4* and the *rps2* mutant backgrounds, respectively, and examined the effects that these mutations had on the constitutively expressed HA-JAZ1. Without pathogen challenge, neither the *HA-JAZ1* mRNA nor the HA-JAZ1 protein was affected by the *npr3 npr4* or *rps2* mutations (Supplementary Fig. 12). However, 4 h after *Psm* ES4326/*avrRpt2* challenge, HA-JAZ1 protein levels were approximately 50% lower in the WT background, but not in the immune receptor mutant *rps2* (Fig. 5b; Supplementary Fig. 13). Importantly, this reduction in JAZ1 level was not seen in the *npr3 npr4* mutant with the WT level of COI1 protein (Fig. 5b; Supplementary Figs 13 and 14). The trigger for this NPR3/4-mediated JAZ1 reduction is SA-dependent, because in the SA-deficient *sid2* mutant, HA-JAZ1 remained stable at 4 h.p.i. by *Psm* ES4326/*avrRpt2* (Fig. 5c). Interestingly, exogenous application of SA was not sufficient to promote the reduction of JAZ1 level through NPR3 and NPR4 indicating that the SA accumulation during ETI is specifically required (Fig. 5d; Supplementary Fig. 15a).

To test whether this NPR3/4-dependent reduction of JAZ1 protein levels during ETI is dependent on the 26S proteasome, we infiltrated MG115 together with *Psm* ES4326/*avrRpt2* and found that addition of MG115 completely blocked the reduction in JAZ1 levels during ETI (Fig. 5b). Quantification of protein levels showed that the JAZ1 protein in the MG115-treated sample accumulated to >100% of the MgSO_4_-treated control (Supplementary Fig. 13), consistent with the observation that a JAZ1 levels were also reduced non-specifically as a result of the inoculation procedure (Supplementary Fig. 16). We further confirmed that mutating both NPR3 and NPR4 did not affect JAZ1 degradation triggered by exogenous application of JA (Supplementary Fig. 15b). These results suggest that NPR3 and NPR4 can specifically promote the degradation of JAZ1 through the activity of the 26S proteasome at the early stage of RPS2-mediated ETI.

### NPR3/4 are required for ETI-triggered *de novo* JA synthesis

To test whether the NPR3/4 are required for *de novo* JA synthesis during ETI (Fig. 2), we measured free SA, SAG, JA and JA-Ile levels in the *npr3 npr4* mutant in response to *Psm* ES4326/*avrRpt2.* We found that in *npr3 npr4*, the total SA level was lower than in WT at 0 h.p.i., whereas the free SA level was less induced compared with WT at 12 h.p.i. (Supplementary Fig. 17). These results could be due to the accumulation of NPR1, a known repressor of SA synthesis^42^, in *npr3 npr4* (ref. 23). Consistent with our hypothesis that *de novo* JA synthesis may be a consequence of early reduction of JAZ levels and induction of JA-responsive genes, we detected significantly lower JA and JA-Ile levels in the *npr3 npr4* mutant in comparison with WT at 12 h.p.i. (Fig. 5e; Supplementary Fig. 17d). These data indicated that NPR3/4 promote *de novo* JA synthesis during RPS2-mediated ETI.

To determine whether the lack of *de novo* JA synthesis and/or signalling is responsible for the compromised ETI in the *npr3 npr4* mutant, we sprayed *npr3 npr4* plants with MeJA at 3.5 h.p.i. with *Psm* ES4326/*avrRpt2*. We used a higher dose of *Psm* ES4326/*avrRpt2* for this experiment, because of the elevated basal resistance in the *npr3 npr4* mutant due to the accumulation of NPR1 (ref. 23). MeJA application recovered resistance in the *npr3 npr4* mutant, but did not further increase resistance in WT (Fig. 5f; Supplementary Fig. 18). The rescue of ETI in *npr3 npr4* by the exogenous application of MeJA is likely through the activity of COI1 protein which is intact in the mutant (Supplementary Fig. 14). This result indicates that NPR3/4-mediated activation of early JA signalling and/or synthesis is essential for RPS2-mediated ETI and the canonical JA pathway is also important for the subsequent signal amplification.

## Discussion

Through this study, we discovered a noncanonical mechanism of activating the JA signalling pathway through the SA receptors NPR3 and NPR4, instead of the canonical JA receptor COI1, in the early stage of ETI induction. This early JA signalling induction event is required for and amplified by the subsequent *de novo* JA synthesis and signalling. However, how the JA signalling pathway contributes to ETI in plants requires further investigation.

SA is a necessary signal for the activation of JA signalling at the early stage of ETI (Fig. 3a), but whether SA is a sufficient signal will require further study because exogenous application of SA is known to repress the JA pathway instead of activating it. SA treatment alone could not lead to the degradation of JAZ1 protein as occurs following inoculation with *Psm* ES4326/*avrRpt2* (Fig. 5d; Supplementary Fig. 15a). ETI may cause *in planta* accumulation of SA to a higher level than that achieved through exogenous application or in a specific subcellular compartment or trigger SA synthesis with a unique kinetic. Alternatively, during ETI, a co-activator may be produced for SA-mediated stimulation of JA signalling.

**Figure 6:**
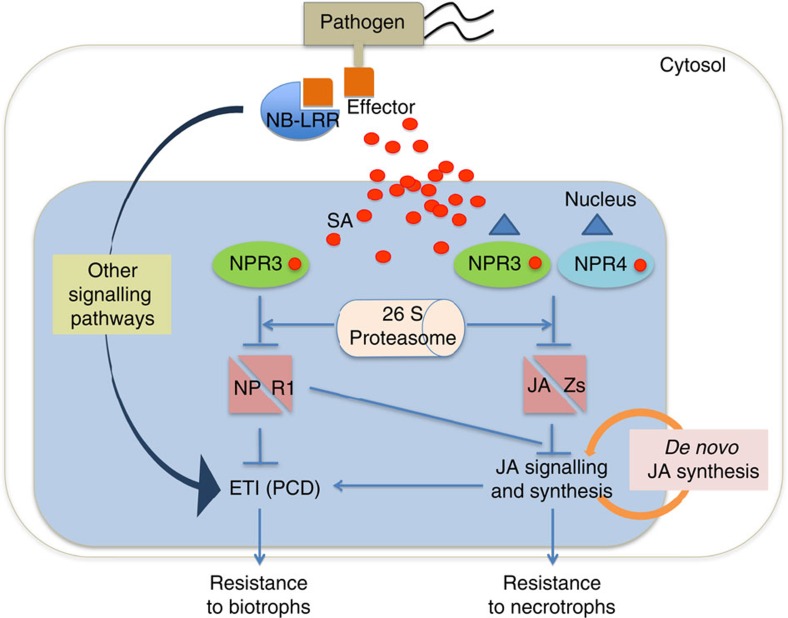
Working model of the interplay between SA and JA during ETI. Activation of an NB-LRR immune receptor in plants by a pathogen effector leads to induction of multiple signalling pathways. A major event of the induction is the accumulation of SA at the infection site. At the high level of SA, NPR3 can interact with its substrate NPR1 to remove its repression on ETI and on crosstalk inhibition of the JA signalling pathway. Both NPR3 and NPR4 can also interact with JAZ proteins in an SA-enhanced manner leading to the degradation of JAZs. This results in activation of *de novo* JA synthesis of JA and amplification of the JA signalling through the canonical pathway. Activation of both SA- and JA-signalling pathways during ETI enables plants to use this PCD-associated defence strategy against biotrophic pathogens without making them vulnerable to necrotrophic pathogens. The blue triangle shape in the graph represents an unknown signal that may affect the degradation of JAZ1 through NPR3 and NPR4.

As summarized in Fig. 6, on ETI induction, multiple signalling pathways are activated. A major event of the induction is the accumulation of SA. The high level of SA at the infection site promotes NPR3-mediated degradation of NPR1 (ref. 23) to remove its repression on ETI (PCD) and alleviate its crosstalk inhibition on JA signalling, meanwhile we propose that degradation of JAZs by NPR3 and NPR4 directly activates JA signalling, which is further amplified by *de novo* JA synthesis. It is intriguing that SA inhibits the NPR4-NPR1 interaction^23^, but stimulates the NPR4-JAZs interaction (Fig. 4c; Supplementary Fig. 8), consistent with the ETI deficient phenotype observed in the *npr4* mutant^23^.

The recovery of ETI resistance in the *npr3 npr4* mutant by the exogenous application of JA (Fig. 5f) indicates that the subsequent JA signal amplification through the canonical pathway is also important for this immune response. This explains the ETI-deficiency observed in the JA synthesis and signalling mutants (Fig. 1).

The synergy between SA and JA during ETI is distinct from the previously reported antagonism^8^,^16^,^43^, which was normally observed in the context of basal resistance^10^,^11^,^15^. Although it is not known how exactly these opposing relationships between the two defence hormones are regulated, the unique interplay between SA and JA during ETI supports the minority opinion^44^ that ETI is not an amplified and prolonged version of basal resistance but rather a distinct immune strategy that is only deployed when basal defence becomes insufficient.

Our study demonstrated a unique functional role for JA in ETI. The cooperation between SA and JA, two defence hormones against biotrophic and necrotrophic pathogens, provides a mechanism by which plants can use PCD as a major defence mechanism against biotrophic pathogens without making them vulnerable to necrotrophic pathogens^10^.

## Methods

### Plant materials

All the mutants used in this research were in the Columbia (Col-0) ecotype background. The mutants *aos*, *sid2*, *npr1*, *npr3 npr4*, *npr1 npr3 npr4*, *rps2*, *coi1-30*, the *35S:HA-JAZ1* line and the *35S:jaz1Δjas* line were described previously^10^,^23^,^45^,^46^. *35S:GFP*, *35S:NPR3-GFP*, *35S:NPR4-GFP* were constructed using the pK7FWG2 vector and transformed to wild type. The *35S:HA-JAZ1* line was crossed with *npr3 npr4*, *sid2* and *rps2* to generate the *35S:HA-JAZ1/npr3 npr4*, *35S:HA-JAZ1/sid2* and *35S:HA-JAZ1/rps2* lines, and crossed with *35S:GFP*, *35S:NPR3-GFP*, *35S:NPR4-GFP* to generate *35S:HA-JAZ1/35S:GFP*, *35S:HA-JAZ1/35S:NPR3-GFP*, *35S:HA-JAZ1/35S:NPR4-GFP*, respectively.

### Quantitative real time PCR

Leaf tissues were collected from 3-week-old plants after the corresponding treatment at the indicated time points and total RNA was extracted using TRIzol Reagent according to the manufacture's instruction. Two microgram of the total RNA sample was treated with TURBO DNA-free (Cat No. AM1907, Invitrogen) to eliminate the genomic DNA contamination. Then cDNA was synthesized by SuperScript III reverse transcriptase (Cat No. 18080-044, Invitrogen) along with oligo-dT primers and analysed by quantitative reverse transcription PCR using SYBR Green Master with the gene specific primers listed in Supplementary Table 1. The level of *UBQ5* was used to normalize the expression of target genes. Data analysis was performed using mixed linear models in the R programming environment^47^. Genotypes and treatments were used as fixed effects and replicate-specific effects were considered random effects. Modelled expression values between genotypes were compared using Student's *t*-test where indicated.

### Split luciferase assay

Split luciferase assay was performed according to a classic method^48^. *JAZs* were cloned into a pDEST vector with the C-terminal half of luciferase (cLuc) and *NPR3* and *NPR4* were cloned into another pDEST vector with the N-terminal half of luciferase (nLuc). They were then individually transformed into *Agrobacterium tumfaciens* GV3101 and transiently co-expressed in one half of each *Nicotianna benthamiana* leaf by infiltration^49^. As a control, the other half of each leaf was co-infiltrated with GV3101 carrying the empty cLuc vector and the NPR3-nLuc or the empty cLuc vector and NPR4-nLuc construct. Two days after inoculation, 1 mM luciferin was sprayed onto the inoculated leaves and chemiluminescence images were taken by a CCD camera 0 and 3 h after 1 mM SA treatment. The fluorescence intensity was quantified using ImageJ.

### Yeast two-hybrid

*JAZ*s and *JAZ1* derivatives were cloned into pGADT7. *NPR3*, *NPR4* and *NPR3* derivatives were cloned into pGBKT7 (ref. 50). These clones were transformed to the *Saccharomyces cerevisiae* strains AH109 and Y187, respectively, according to the Clontech yeast transformation protocol. After mating, diploid cells were selected on SD-Leu-Trp (SD-LW) plates. Single-positive colonies from each of the SD-LW plates were grown in the SD-LW liquid media for 1 day and then used for testing the interaction on SD-Leu-Trp-His-Ade (SD-LWHA) plates and SD-LWHA+100 μM SA plates.

### Co-immunoprecipitation and pull down assays

For co-IP in *N. benthamiana, NPR3* and *NPR4* were cloned into the pK7FWG2 vector to create a C-terminal GFP fusion and *JAZ1* was cloned into pEG203 to create an N terminal Myc fusion^51^,^52^. The NPR3-GFP, NPR4-GFP or GFP were co-expressed with Myc-JAZ1 in leaves of *N. benthamiana* for 2 days. Total proteins were extracted using the extraction buffer consisting 50 mM Tris (pH 7.5), 150 mM NaCl, 0.1% Triton X-100, 0.2% NP-40, 40 μM MG115, and the protease inhibitor cocktail. 100 μM SA was added to the protein samples and then incubated with GFP-Trap_A beads (Cat No. gta-10, ChromoTek) for 2 h at 4 °C. For co-IP in *Arabidopsis*, 3-week-old plants of *35S:HA-JAZ1/35S:GFP*, *35S:HA-JAZ1/35S:NPR3-GFP*, *35S:HA-JAZ1/35S:NPR4-GFP* were infiltrated with *Psm* ES4326/*avrRpt2* at OD_600nm_=0.2 together with 40 μM MG115, and samples were collected 4 h.p.i. Then total proteins were extracted using the extraction buffer and incubated with GFP-Trap_A beads for 2 h at 4 °C. After GFP-Trap_A beads were washed three times with the extraction buffer in the co-IPs performed in both *N. benthamiana* and *Arabidopsis,* proteins were eluted and denatured by heating in the protein loading buffer containing 100 mM dithiothreitol at 95 °C for 5 min before loading onto SDS–PAGE gels. The HA-JAZ1 or Myc-JAZ1 protein that co-precipitated with the GFP-tag proteins was detected by western blotting^53^ using the Myc (Cat No. sc-40, Santa Cruz Biotechnology; dilution, 1:1,000) or HA (Cat No. MMS-101P, Covance; dilution, 1:1,000) antibody. The HA-JAZ1, Myc-JAZ1 and GFP, NPR3-GFP or NPR4-GFP input levels were also measured by using HA, Myc or GFP (Cat No. JL-8, Living Colours; dilution, 1:2,000) antibody, respectively.

For the pull down assay, GST and GST-JAZ1 (ref. 54) were expressed in *E. coli* for 5 h at 25 °C after IPTG induction. The NPR3-GFP and NPR4-GFP were transiently expressed in the *NahG N. benthamiana* transgenic line^55^ for 2 days, and treated with or without 1 mM SA or 100 μM MeJA 3 h before sample collection. After purification using Glutathione magnetic beads (Cat No. 88822, Thermo Scientific), GST and GST-JAZ1 were incubated with protein extract of NPR3-GFP or NPR4-GFP separately for 2 h at 4 °C. Then beads were washed three times with the extraction buffer, and proteins were denatured by heating in the protein loading buffer containing 100 mM dithiothreitol at 95 °C for 5 min before loading onto SDS-PAGE gels. The NPR3-GFP and NPR4-GFP were detected by western blotting using the GFP antibody. The GST and GST-JAZ1 input levels were shown by Coomassie blue staining. Full versions of cropped blots are shown in Supplementary Fig. 19.

### Hormone measurement

Phytohormones were extracted and quantified according to the method of Zeng *et al*.^56^ with some modifications. Four to six of the 3rd and 4th true leaves of 3-week-old plants (∼50 mg) were harvested before or after *Psm* ES4326/*avrRpt2* infiltration at OD_600nm_=0.01 at corresponding times. Samples were extracted at 4 °C overnight (∼16 h) using 0.45 ml of ice cold methanol:water (80:20 v/v) containing 0.1% formic acid, 0.1 g l^−1^ butylated hydroxytoluene (BHT) spiked with abscisic acid (ABA)-d_6_ (100 nM) as an internal standard. Filtered plant extracts were injected onto a C18 column, and a gradient method starting with 9:1 (v/v) of solvent A (0.1% aqueous formic acid) and solvent B (100% methanol) and increasing linearly to 100% solvent B was used for separation. For mass spectrometry, the capillary voltage, cone voltage and extractor voltage were set to 3.5 kV, 25 and 5 V, respectively. Desolvation gas and cone gas were set to flow rates of 600 and 50 l h^−1^, respectively. Selected ion monitoring (SIM) was conducted in the negative electrospray channel for salicylic acid (SA; *m*/*z* 137>93), SA glucoside (SAG; *m*/*z* 299.1>137), abscisic acid (ABA; *m*/*z* 263.1>153.1), jasmonic acid (JA; *m*/*z* 209.1>59), JA-isoleucine (JA-Ile; *m*/*z* 322.2>130.1) and the internal ABA-d_6_ standard (*m*/*z* 269.1>159.1). Quan-Optimize software was used to determine the parent>daughter SIM pairs used as well as the optimal source cone and collision energy voltages for each compound monitored. QuanLynx v4.1 software was used to determine analyte responses based on peak area integrations relative to the internal standard. Analytes were then quantified based on standard curves to determine the concentrations (nM), which were converted to nanogram using the molecular weight of the compound and the extraction volume and normalized by sample fresh weight (FW) in gram. SAG was quantified based on the SA standard curve.

### Pathogen infection

*Psm* ES4326/*avrRpt2* was used to induce gene expression by infiltration into the 3rd and 4th true leaves of 3-week-old plants at OD_600nm_=0.2 and samples were collected 4 h.p.i. The ion leakage assay was performed^57^ with six leaf discs suspended in 6 ml of de-ionized water were used to measure the conductivity at OD_600nm_=0.01. To check the effect of JA during ion leakage assays, 100 μM MeJA or water was applied 3.5 h.p.i., and the leaf discs were collected 30 min later. To measure the bacterial growth, *Psm* ES4326/*avrRpt2* was infiltrated into the 3rd and 4th true leaves of 3-week-old plants at indicated dosages, and *in planta* bacteria counts were monitored according to Cao *et al*.^1^.

### Protein degradation

To analyse JAZ1 protein stability during ETI, 10 mM MgSO_4_, *Psm* ES4326/*avrRpt2* or *Psm* ES4326/*avrRpt2* with 40 μM MG115 was infiltrated into the 3rd and 4th leaves of 3-week-old plants at OD_600nm_=0.2. After 4 h, 0.2 g of the infiltrated leaves were collected for each sample. To measure the JAZ1 stability affected by SA in soil growing plants, 1 mM SA were sprayed on 3-week-old plants for 4 h. For the time course assay of JAZ1 stability, 2-week-old seedlings on MS plates were sprayed with 200 μg ml^−1^ CHX (Mock), 1 mM SA plus 200 μg ml^−1^ CHX (SA) or 100 μM MeJA plus 200 μg ml^−1^ CHX (MeJA), and samples were collected at the corresponding times. Total protein was extracted using the extraction buffer mentioned above and denatured by heating at 95 °C for 5 min after adding the protein loading buffer containing 100 mM dithiothreitol. The HA-JAZ1 protein level was detected by western blotting using the HA antibody. β-Tubulin was also detected as an internal control using the β-Tubulin antibody (Cat No. sc-166729, Santa Cruz Biotechnology; dilution, 1:2,000)^58^. Full versions of cropped blots are shown in Supplementary Fig. 19.

### Statistics

All data analyses were done using GraphPad Prism 6.00. Data are presented as mean±s.d. The Student's *t*-test was used to determine statistical significance for all pairwise comparisons and two-way analysis of variance (ANOVA) for data of the pathogen growth. The two-way ANOVA is a better statistical test for data with two independent variables. In our experiments, the ordinary two-way ANOVA was performed with no multiple comparisons and used colony forming units (the log_10_ c.f.u. in the *y* axis) as dependent variables; days (treatments) after infiltration and genotypes as independent variables. Data distribution in this study was assumed to be normal, but not formally tested. The sample sizes were similar to those generally employed in the field.

### Data availability

The authors declare that all data supporting the findings of this study are available within the article and its Supplementary Information files or are available on request from the corresponding authors.

## Additional information

**How to cite this article:** Liu, L. *et al*. Salicylic acid receptors activate jasmonic acid signalling through a non-canonical pathway to promote effector-triggered immunity. *Nat. Commun.* **7,** 13099 doi: 10.1038/ncomms13099 (2016).

## Supplementary Material

Supplementary Information Supplementary Figures 1 - 19 and Supplementary Table 1
