# Supplementary material for: Salicylic acid receptors activate jasmonic acid signalling through a non-canonical pathway to promote effector-triggered immunity
Source: Nat Commun. 2016 Oct 11;7:13099. doi: 10.1038/ncomms13099 (PMC5062614; doi:10.1038/ncomms13099)
Supplement: Supplementary Information — Supplementary Figures 1 - 19 and Supplementary Table 1 [file ncomms13099-s1.pdf]

Supplementary Figures

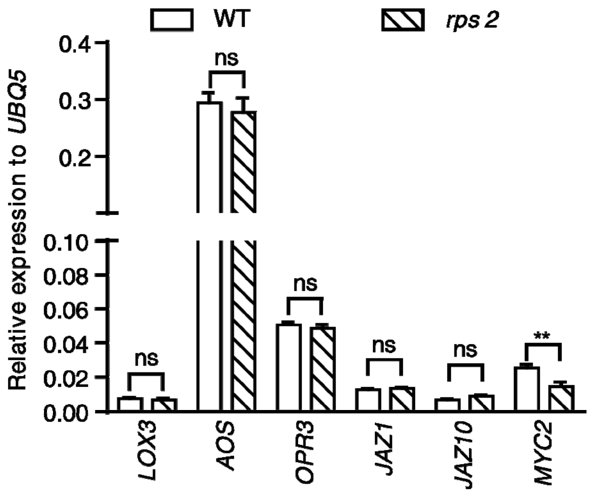

Supplementary Figure 1. Basal expression of JA-responsive genes in WT and *rps2*. Samples were collected from wild type (WT) and the *rps2* mutant without treatment. qRT-PCR was performed on *LOX3*, *AOS*, *OPR3, JAZ1*, *JAZ10*, and *MYC2* using *UBQ5* as a reference. Data from three biological replicates were combined using linear mixed-effects model, and then are shown as mean ± SD. **, $p < 0.01$; ns, no significant difference.

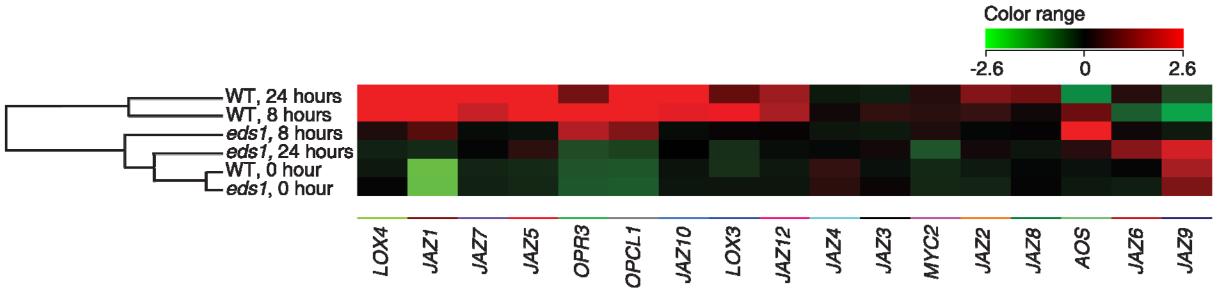

Supplementary Figure 2. Expression levels of JA-responsive genes during RPS4-mediated immune response. GSE50019 (http://www.ncbi.nlm.nih.gov/geo/query/acc.cgi?acc=GSE50019) was the array used for analysis in this study**.** Plants expressing *35S:RPS4-HS* in WT (WT) and the *eds1*mutant (*eds1*) defective in RPS4 signalling, were grown at 28 °C for 3.5 weeks, and subsequently shifted to 19 °C to rapidly turn on the RPS4-mediated and EDS1-dependent immune response. 0 hour, before the shift; 8 and 24 hours, 8 and 24 hours after the shift.

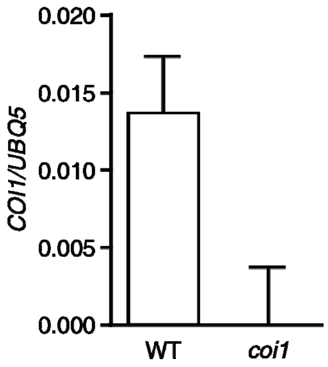

Supplementary Figure 3. Expression level of *COI1* mRNA in WT and the *coi1* mutant. The qRT-PCR was performed on the same cDNA samples used in Fig. 3d. Data from three biological replicates were combined using linear mixed-effects model, and then are shown as mean ± SD.

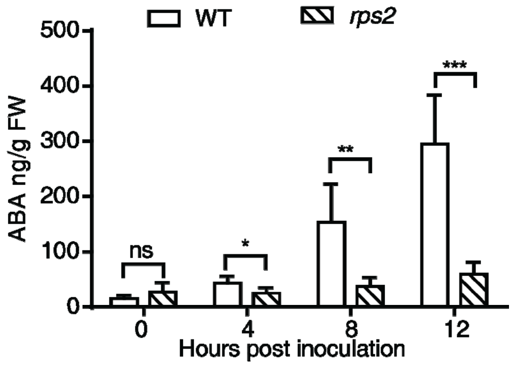

Supplementary Figure 4. The ABA level in WT and *rps2* during ETI. Three-week-old WT and *rps2* plants were infiltrated with *Psm* ES4326/*avrRpt2* at OD_{600nm} = 0.01. Samples were collected at 0, 4, 8, 12 hpi. Significant difference was detected using Student's *t*-test. Data are shown as mean ± SD (n = 5-6 biological replicates). *, $p < 0.05$; **, $p < 0.01$; ***, $p < 0.001$; ns, no significant difference.

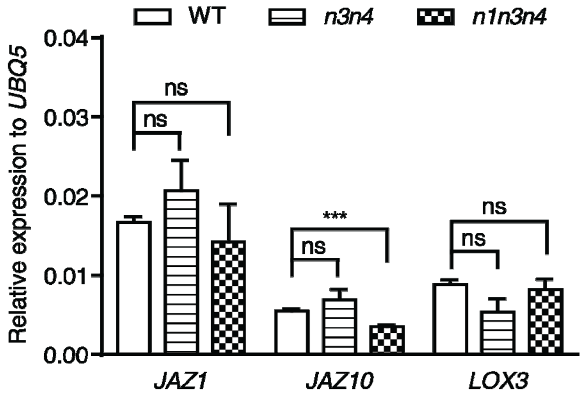

Supplementary Figure 5. Basal expression of JA-responsive genes in WT, *npr3 npr4* and *npr1 npr3 npr4* mutants. Samples were collected from wild type (WT), *npr3 npr4* (*n3n4*) and *npr1 npr3 npr4* (*n1n3n4*) mutants without treatment. qRT-PCR was performed on *JAZ1*, *JAZ10, LOX3* using *UBQ5* as a reference. Data from three biological replicates were combined using linear mixed-effects model, and then are shown as mean ± SD. ***, $p < 0.001$; ns, no significant difference.

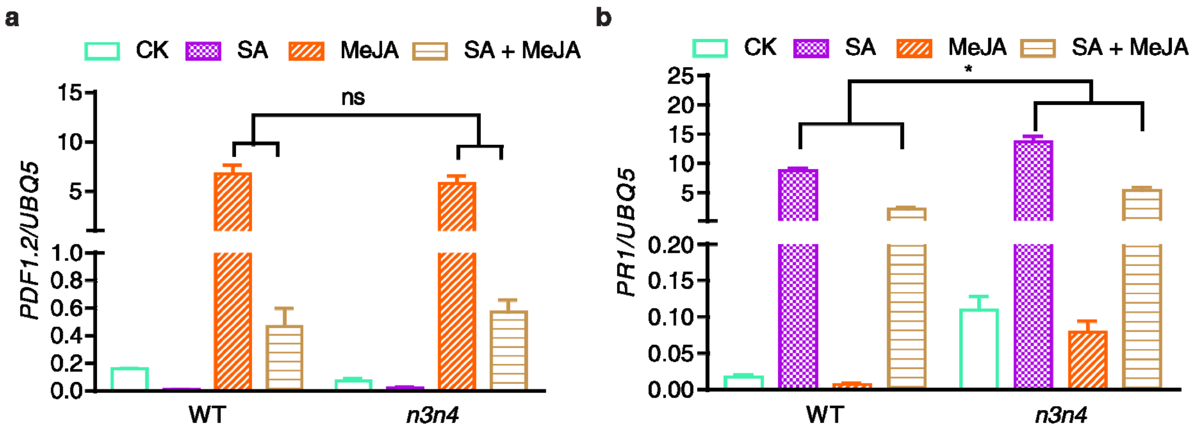

Supplementary Figure 6. The expressions of *PDF1.2* and *PR1* in *npr3 npr4* after SA, JA or SA plus JA treatment. Samples were collected 24 hours after being sprayed with 0.2% EtOH (Mock), 1 mM SA (SA), 20 µM MeJA (MeJA) or both hormones (SA + MeJA). The expression of (**a**) *PDF1.2* and (**b**) *PR1* was measured. Data from three biological replicates were combined using linear mixed-effects model, and then are shown as mean ± SD. *, $p < 0.05$; ns, no significant difference.

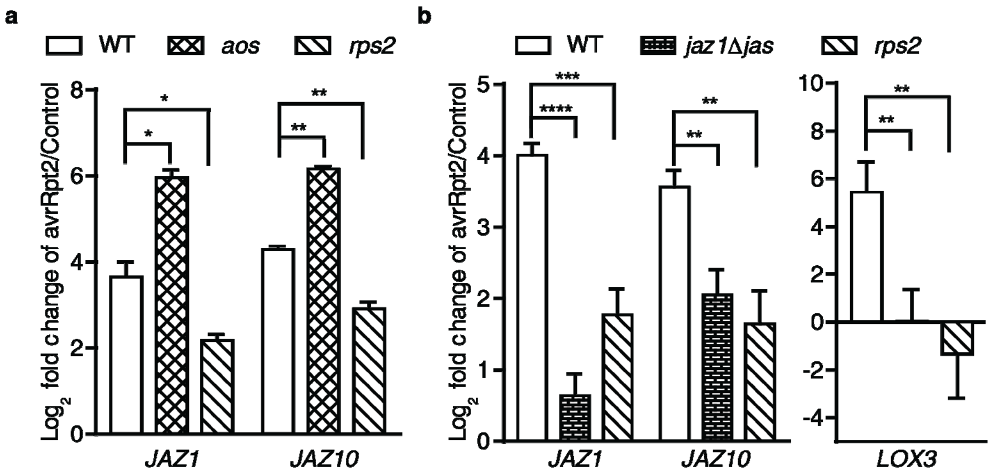

Supplementary Figure 7. ETI-mediated early induction of JA-responsive genes is dependent on the degradation of JAZs, but independent on the JA biosynthetic gene *AOS*. Leaves from (**a**) WT, *aos,* and *rps2* or (**b**) WT, *jaz1Δjas,* and *rps2* were harvested 4 hpi with *Psm* ES4236/*avrRpt2* at OD_{600nm} = 0.2 (avrRpt2) or 10 mM $MgSO_4$ (Control). qRT-PCR was performed on *JAZ1*, *JAZ10* with *UBQ5* as a reference. Data from two (a) or three (b) biological replicates were combined using linear mixed-effects model. Significant difference was detected by Student's *t*-test. Data are shown as mean ± SD. *, $p < 0.05$; **, $p < 0.01$; ***, $p < 0.001$; ****, $p < 0.0001$.

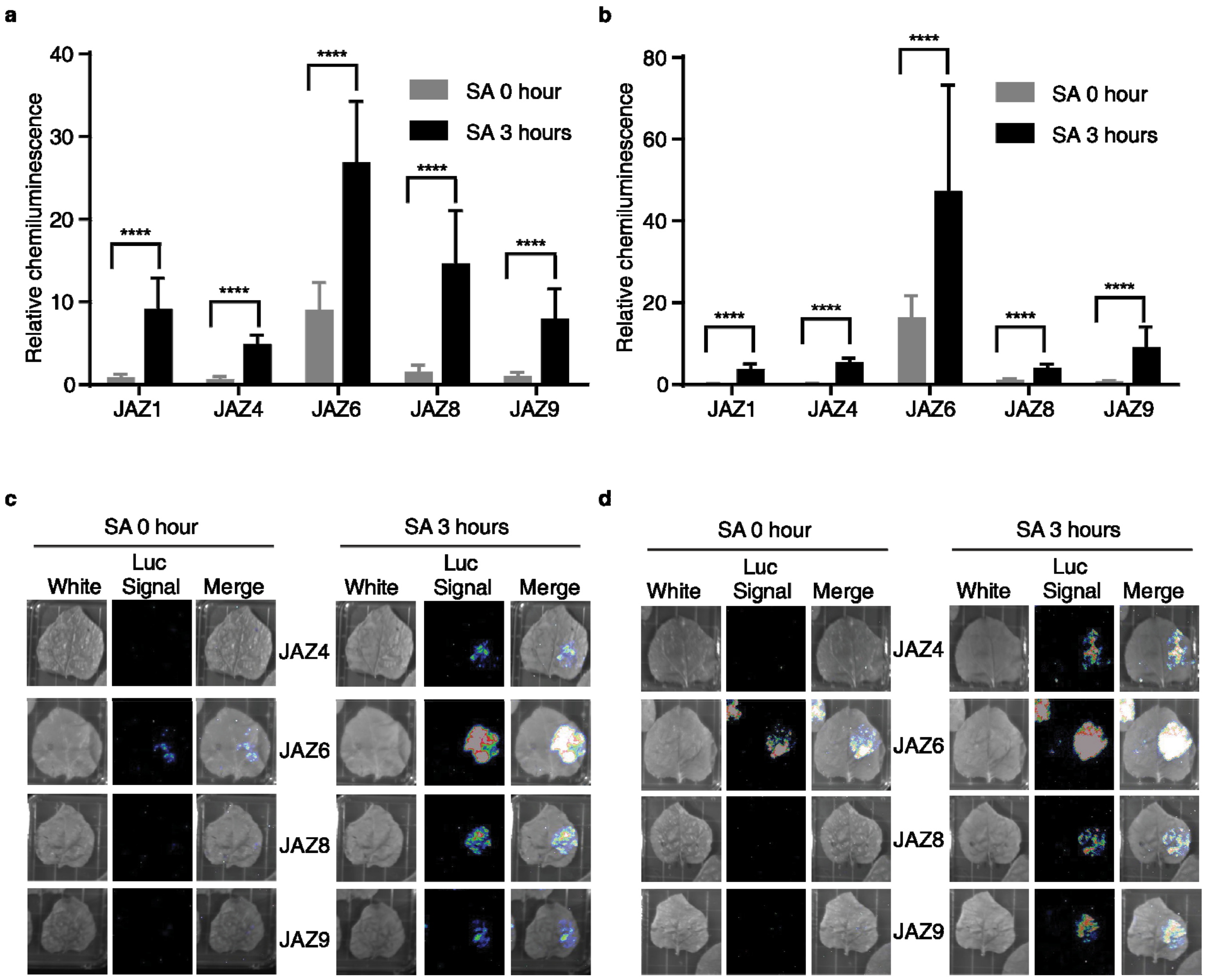

Supplementary Figure 8. Split luciferase assay of interactions between JAZs and NPR3 or NPR4 proteins. Quantification of split luciferase data in Fig. 4c and panels c and d of this figure using ImageJ. (**a**) The interactions between NPR3 and JAZs and (**b**) the interactions between NPR4 and JAZs. Data are shown as mean ± SD (n = 4 biological replicates). All experiments were repeated three times with similar results. Significant difference was detected by Student's *t*-test. ****, $p < 0.0001$. (**c**) The representative leaves for the split luciferase assay of NPR3 and JAZs interaction in (a). (**d**) The representative leaves for the split luciferase assay of NPR4 and JAZs interaction in (b).

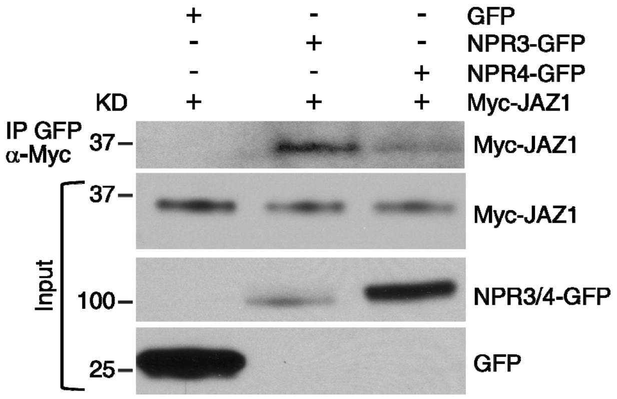

Supplementary Figure 9. Co-immunoprecipitation (co-IP) of JAZ1 using NPR3 or NPR4 in *N. benthamiana*. The Myc-JAZ1 was co-expressed with GFP, NPR3-GFP, or NPR4-GFP individually in *N. benthamiana* leaves for 2 days, and the co-IP assay was carried out using the GFP-Trap®_A beads for 2 hours at 4 °C with a final concentration of 100 μM SA added to the co-IP solution. The Myc-JAZ1 protein was detected by western blotting using the Myc antibody. The NPR3-GFP, NPR4-GFP or GFP protein levels were measured using the GFP antibody.

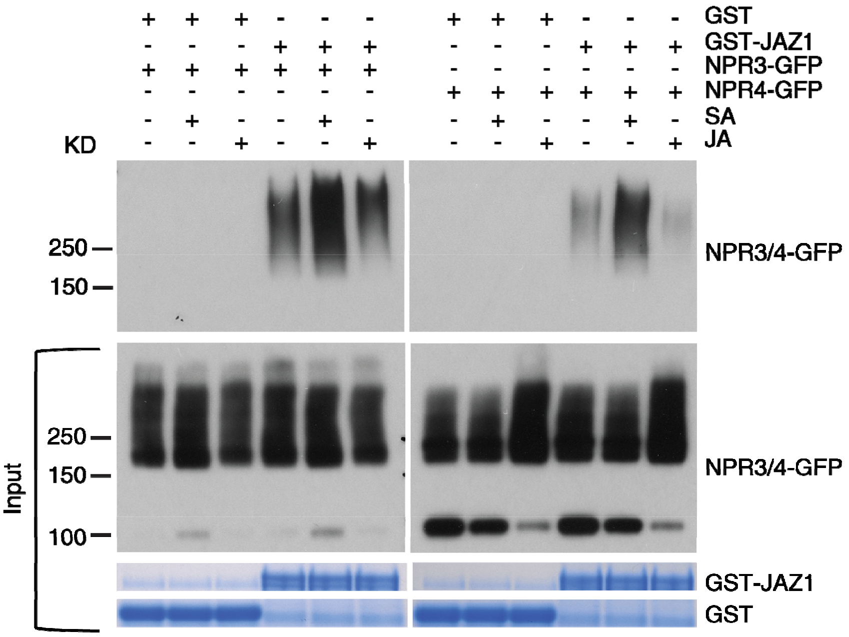

Supplementary Figure 10, Pull down assay of NPR3 or NPR4 by JAZ1 in response to SA or JA treatment. NPR3-GFP and NPR4-GFP were transiently expressed in leaves of *N. benthamiana* carrying the *NahG* transgene, and treated with corresponding hormones 3 hours before sample collection. GST and GST-JAZ1 were expressed in *E.coli*. The pull down assays were performed by adding NPR3-GFP and NPR4-GFP protein extracts to purified GST and GST-JAZ1 on Glutathione magnetic beads. NPR3-GFP and NPR4-GFP were measured by western blotting using the GFP antibody. The GST and GST-JAZ1 input levels were shown by Coomassie blue staining. All experiments were repeated three times with similar results.

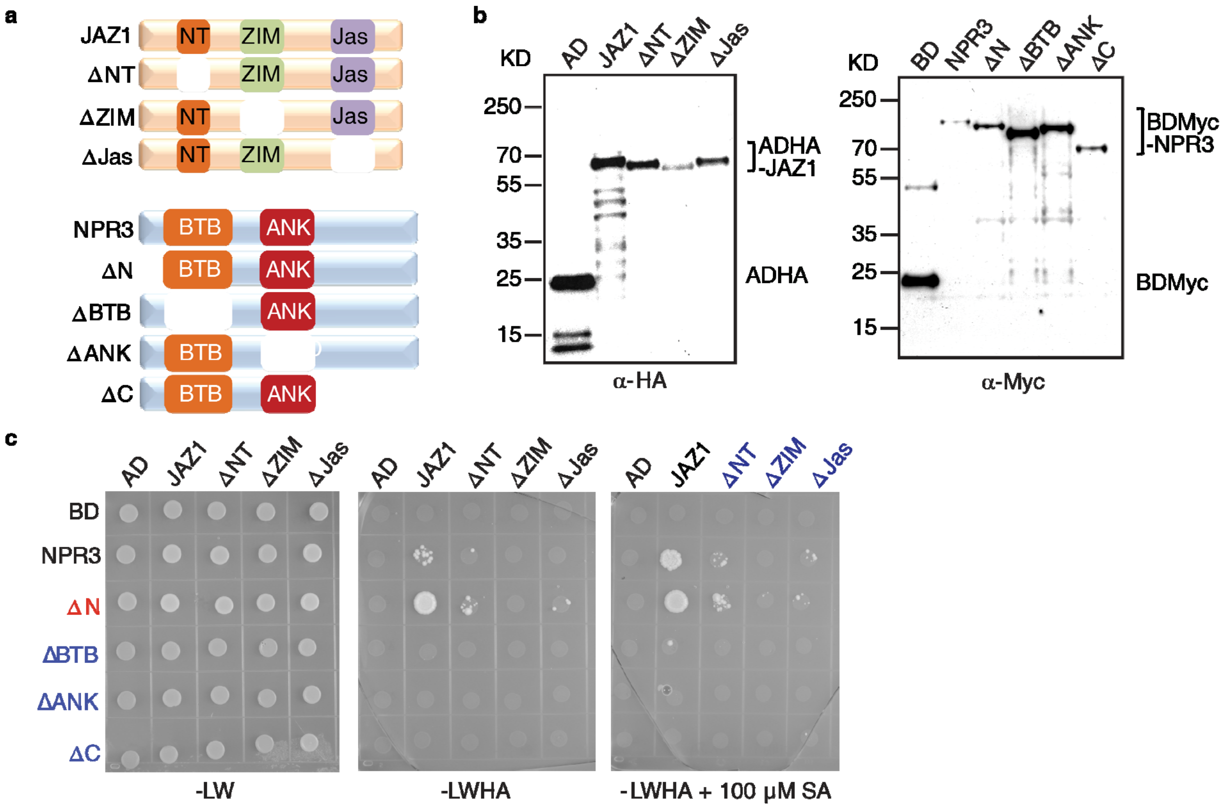

Supplementary Figure 11. Identification of domains required for NPR3-JAZ1 interaction using the yeast two-hybrid assay. (**a**) Schematic representations of NPR3, JAZ1 and their deletion constructs. The protein interaction domains are indicated with different colours and the deleted domains are left as blanks. NT, N-terminal domain; ZIM, zinc-finger expressed in inflorescence meristem domain; Jas, jasmonate-associated domain; BTB, Bric-a-Brac, Tramtrack, Broad-complex domain; ANK, the ankyrin-repeat domain. (**b**) The protein expression levels of the Y2H constructs. Yeast total proteins were extracted from equal amounts of cell culture. Western blots were performed with Myc and HA antibodies to check the expression of NPR3, JAZ1 and their derivatives separately. (**c**) Y2H assay between NPR3 and JAZ1 derivatives. JAZ1 and its derivatives were fused with the AD domain, and NPR3 and its derivatives with the BD domain. After mating, the diploid cells were used for testing interaction on different plates. Compared to WT form NPR3 and JAZ1, deletions showing reduced

interactions are labelled blue and enhanced interactions are labelled red. All experiments were repeated three times with similar results.

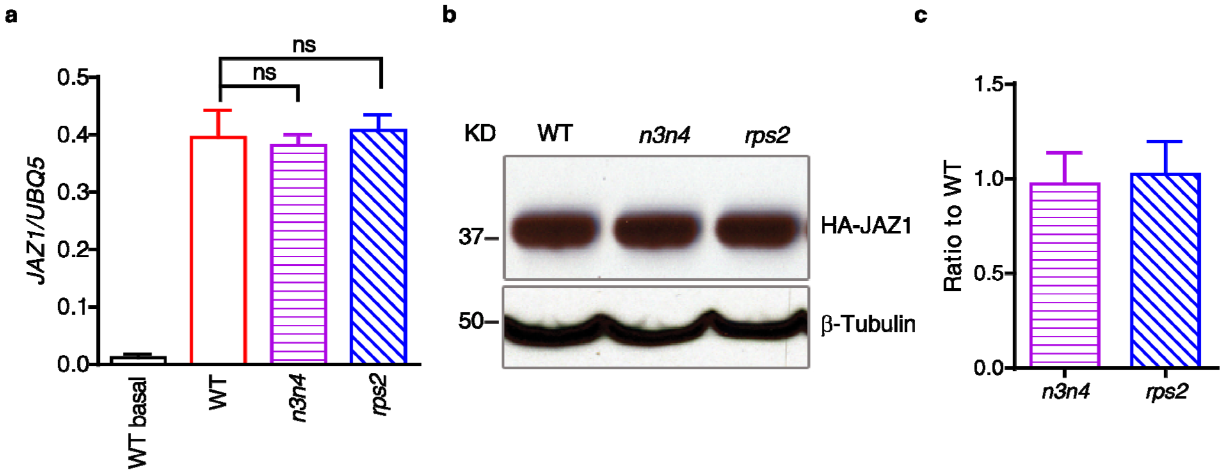

Supplementary Figure 12. JAZ1 mRNA and protein levels in WT, *npr3 npr4,* and *rps2* backgrounds. (**a**) The mRNA levels of *JAZ1* in untransformed plants (WT basal), *35S:HA-JAZ1* transgenic line in wild-type (WT), *npr3 npr4* (*n3n4*) and *rps2* (*rps2*) backgrounds were measured by qRT-PCR using *UBQ5* as an expression reference. Data from three biological replicates were combined using linear mixed-effects model, and then are shown as mean ± SD. Significant difference was detected by Student's *t*-test. ns, no significant difference. (**b**) The HA-JAZ1 protein levels in the *35S:HA-JAZ1* transgenic line in wild-type (WT), *npr3 npr4* (*n3n4*) and *rps2* (*rps2*) backgrounds were measured by western blotting using the HA antibody. The β-Tubulin levels were also detected as a control. (**c**) The HA-JAZ1 protein levels detected in *35S:HA-JAZ1* in *npr3 npr4* (*n3n4*) and *rps2* backgrounds were normalized to that in the WT background. Data are shown as mean ± SD (n = 3 biological replicates).

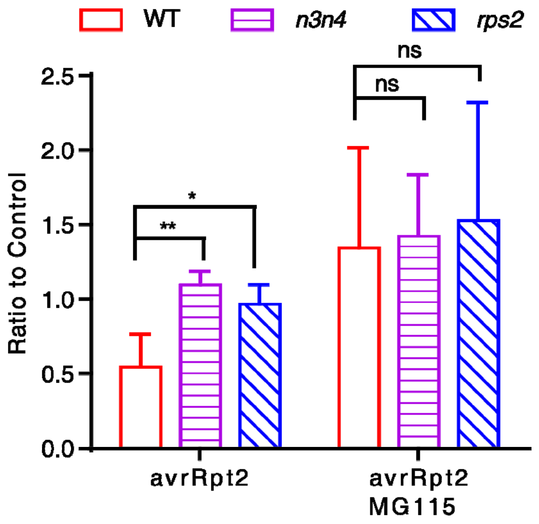

Supplementary Figure 13. Quantification of HA-JAZ1 protein levels in *35S:HA-JAZ1* in WT, *npr3 npr4* and *rps2* backgrounds after ETI induction. The HA-JAZ1 protein levels in *Psm*ES4326/*avrRpt2*-treated (avrRpt2) and *Psm*ES4326/*avrRpt2* with MG115-treated (avrRpt2 MG115) samples were normalized to those of the 10 mM $MgSO_4$ -treated (Control) samples. Experiments were performed as described in Fig. 5b. Data are shown as mean ± SD (n = 4 biological replicates). Significant difference was detected by Student's *t*-test. *, $p < 0.05$; **, $p < 0.01$; ns, no significant difference.

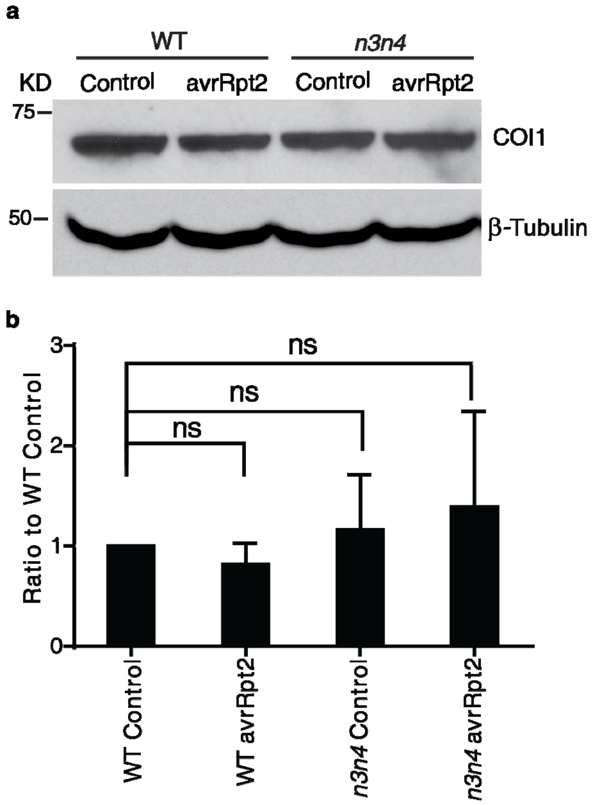

Supplementary Figure 14. The COI1 protein levels in *35S:HA-JAZ1* in WT and *npr3 npr4* backgrounds after ETI induction. COI1 protein levels were determined in *35S:HA-JAZ1* (WT), and *35S:HA-JAZ1/npr3 npr4* (*n3n4*) at 4 hpi with 10 mM $MgSO_4$ (Control), or *Psm* ES4326/*avrRpt2* (avrRpt2). (**a**) COI1 was detected by a COI1 antibody (Cat No. AS12 2637, Agrisera; dilution, 1:1,000), and β-Tubulin was detected as an internal control. KD, kilodalton. (**b**) Quantification of the COI1 protein levels in (a). The COI1 protein levels were normalized to those in WT Control samples. Data are shown as mean ± SD (n = 3 biological replicates). Significant difference was detected by Student's *t*-test. ns, no significant difference.

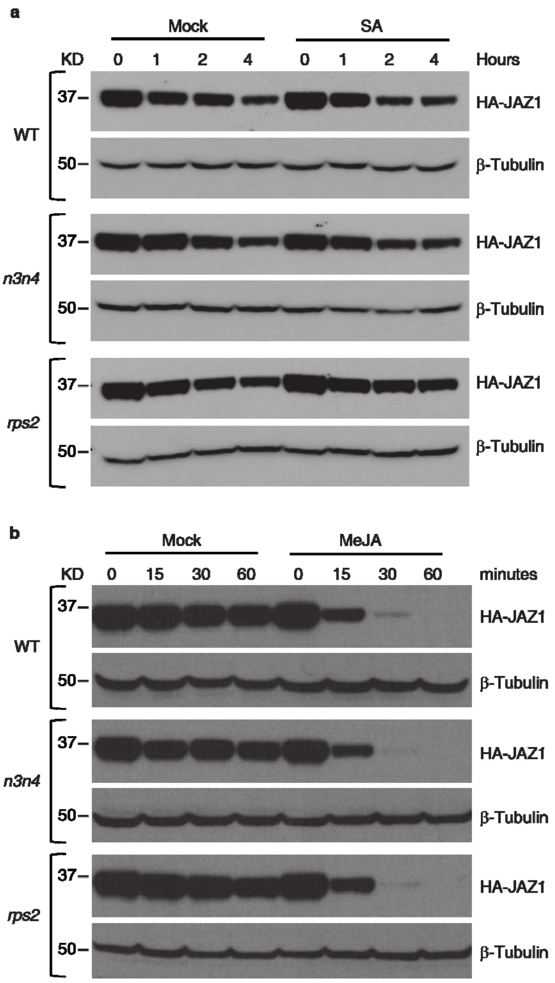

Supplementary Figure 15. The stability of JAZ1 after SA or JA treatment in *35S:HA-JAZ1* in WT, *npr3 npr4* and *rps2* backgrounds. Two-week-old seedlings on MS plates were sprayed with 200 µg/ml CHX (Mock), 1 mM SA plus 200 µg/ml CHX (SA) or 100 µM MeJA plus 200 µg/ml CHX (MeJA) for corresponding time points. (**a**) SA treatment. (**b**) JA treatment. The HA-JAZ1 protein levels were determined in WT, *npr3 npr4* (*n3n4*) and the *rps2* background by western blotting using an HA antibody. β-Tubulin served as a loading control. All experiments were repeated twice with similar results. KD, kilodalton.

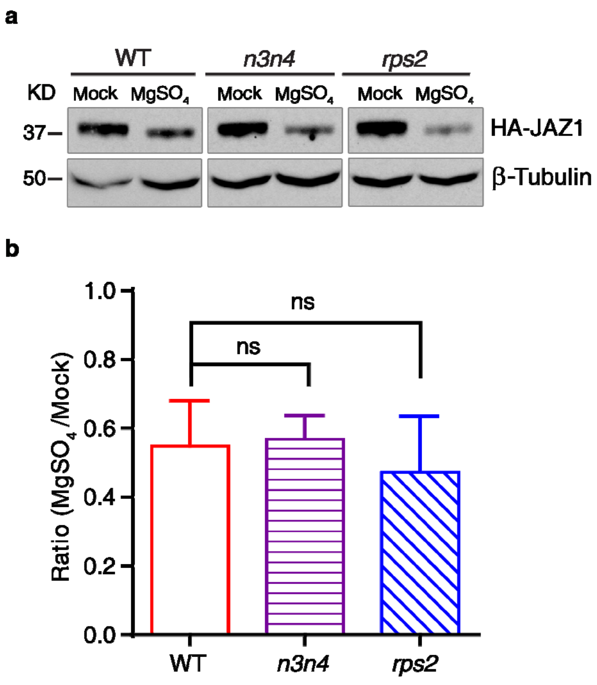

Supplementary Figure 16. HA-JAZ1 protein levels in *35S:HA-JAZ1* in WT, *npr3 npr4* and *rps2* backgrounds after $MgSO_4$ infiltration. (**a**) HA-JAZ1 protein levels were determined in WT, *npr3 npr4* (*n3n4*) and the *rps2* mutant 4 hours after non-treatment (Mock) or $MgSO_4$ infiltration ($MgSO_4$). The western blots were performed using the HA antibody. β-Tubulin served as a loading control. KD, kilodalton. (**b**) The protein levels in $MgSO_4$ infiltrated samples were normalized to those of the non-treated samples. Data are shown as mean ± SD (n = 3 biological replicates). Significant difference was detected by Student's *t*-test. ns, no significant difference.

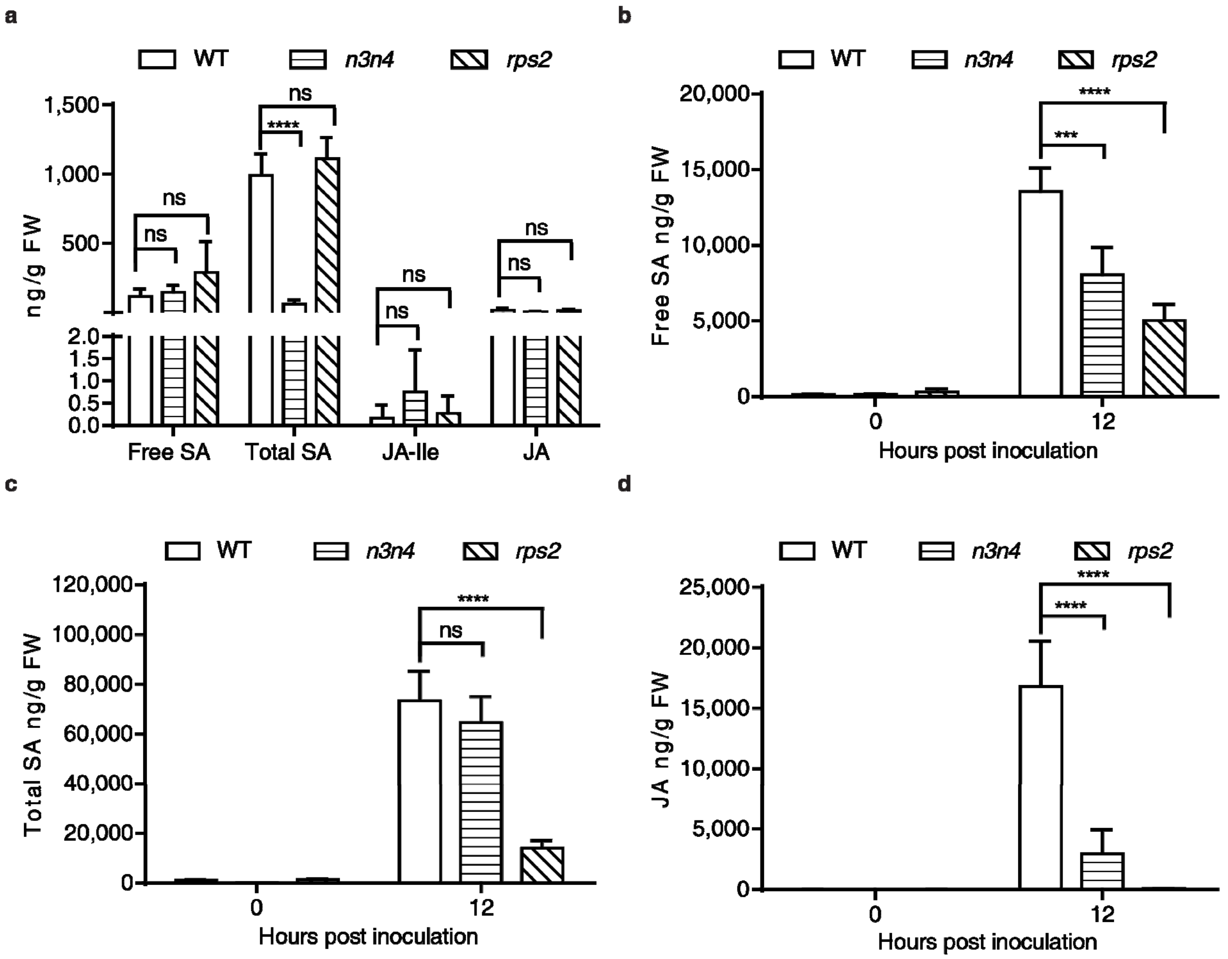

Supplementary Figure 17. The hormone levels in WT, *npr3 npr4* and *rps2* before and after ETI induction. Three-week-old WT, *npr3 npr4* (*n3n4*) and *rps2* plants were infiltrated with *Psm* ES4326/*avrRpt2* at OD_{600nm} = 0.01. Samples were collected at 0 and 12 hpi. (**a**) The basal levels of free SA, total SA, JA-Ile and JA in WT, *n3n4* and *rps2*. The levels of (**b**) free SA, (**c**) total SA, and (**d**) JA in WT, *n3n4* and *rps2* at 0 and 12 hpi with *Psm* ES4326/*avrRpt2*. Data are shown as mean ± SD (n =5-6 biological replicates). Significant difference was detected using Student's *t*-test. *, $p < 0.05$; **, $p < 0.01$; ****, $p < 0.0001$; ns, no significant difference. All experiments were repeated three times with similar results.

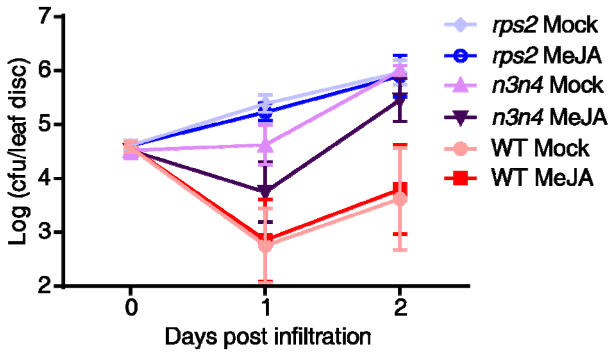

Supplementary Figure 18. *Psm* ES4326/*avrRpt2* growth in WT, *npr3 npr4* and *rps2* with MeJA treatment. Plants were infiltrated with *Psm* ES4326/*avrRpt2* at OD_{600nm} = 0.01, and 3.5 hours later water (Mock) or 100 μM MeJA (MeJA) was sprayed. Pathogen growth were measured at 0, 1, 2 days post inoculation with *Psm* ES4326/*avrRpt2.* Data are shown as mean ± SD (n = 8 biological replicates). This experiment was repeated twice with similar results.

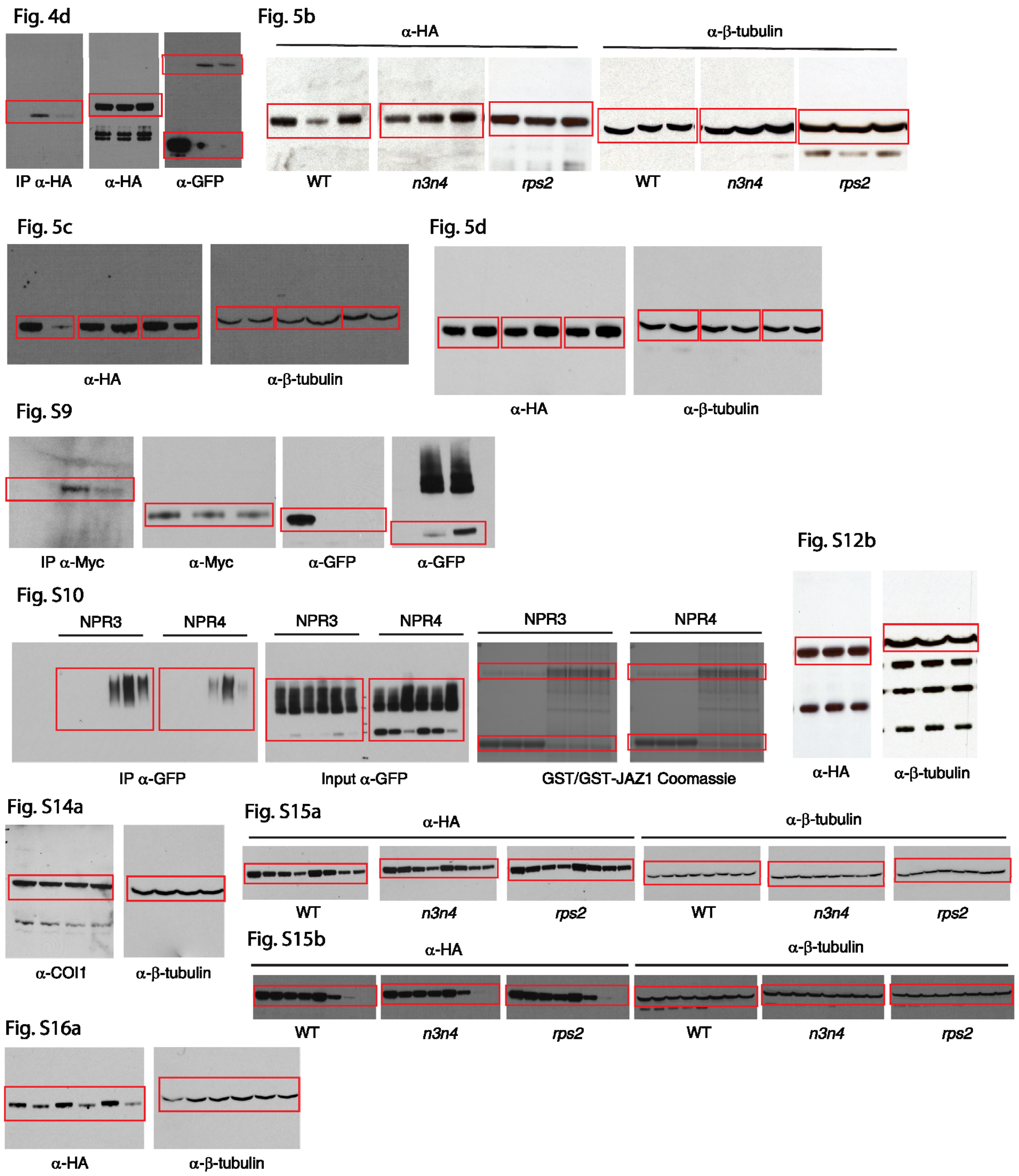

Supplementary Figure 19. Full scan data of immunoblots and Coomassie stained images. Full blots are provided unless already shown in the figures themselves.

Supplementary Table

Supplementary Table 1. The primers used in this study.

Primer	Sequence (5' to 3')
JAZ1-qPCR-For	TTCTGAGTTCGTCGGTAGCC
JAZ1-qPCR-Rev	AGGCTTGCATGCCATTCCTA
AOS-qPCR-For	GTGAAATGCTTTACGGTTATC
AOS-qPCR-Rev	ACCAAAACAACAAAATCCTTAC
MYC2-qPCR-For	CGGCGGAGCTGGAGATTTAT
MYC2-qPCR-Rev	TAGACGGGTCGTTCTCACCT
OPR3-qPCR-For	ACCCACTATCACTCGGGCTA
OPR3-qPCR-Rev	CGTGGTAGCGAGGTTGTGTA
JAZ10-qPCR-For	CGCCAGGTCTAGTACCGAAC
JAZ10-qPCR-Rev	TGCTGCTTCATTAGCGACCT
LOX3-qPCR-For	TCATCACTTGTCTCGTCGGC
LOX3-qPCR-Rev	CGACGCCGGATTTGGTTTTT
PR1-qPCR-For	CTCATACACTCTGGTGGG
PR1-qPCR-Rev	TTGGCACATCCGAGTC
PDF1.2-qPCR-For	TTGCTGCTTTCGACGCA
PDF1.2-qPCR-Rev	TGTCCCACTTGGCTTCTCG
COI1-qPCR-For	TGATGATGTCATCGAGCAAG
COI1-qPCR-Rev	ATGCTCTCTCGTCTCGGAAT
UBQ5-qPCR-For	GTAAACGTAGCTCAGTCCA
UBQ5-qPCR-Rev	GACGCTTCATCTCGTCC
JAZ1-ΔNT-For	AGCCGGAGATTTACTGGGAAGGTCAATGGAACTTTAGGCAACTC
JAZ1-ΔNT-Rev	GTTGCCTAAAGTTCCATTGACCTTCCCAGTAAATCTCCGGC

JAZ1-ΔZIM-For	CAGACTACCAGATCTGTGAAAAGCAAAGGCACCGCTAATAGC
JAZ1-ΔZIM-Rev	TATTAGCGGTGCCTTTGCTTTTCACAGATCTGGTAGTCTGTG
JAZ1-ΔJas-For	CCCCAACACCATTGACAGAATTATGCGATCCAGCCAAAGCGTC
JAZ1-ΔJas-Rev	CGCTTTGGCTGGATCGCATAATTCTGTCAATGGTGTTGGGGAG
NPR3-ΔN-For	GGGGACAAGTTTGTACAAAAAAGCAGGCTTATACAGTGATGCAGAGATCATTGTTG
NPR3Rev	GGGGACCACTTTGTACAAGAAAGCTGGGTCTGTTGTGTTGTGCAGGTCATCTC
NPR3-For	GGGGACAAGTTTGTACAAAAAAGCAGGCTTAATGGCTACTTTGACTGAGCCATC
NPR3-ΔC-Rev	GGGGACCACTTTGTACAAGAAAGCTGGGTCTGCGCTACGTCCGTCAGATGTAAAC
NPR3-ΔBTB-For	CTTCTTAGTAATTCAGATTGTGATTGTAACTTTGTGGAGAAGACCCTTG
NPR3-ΔBTB-Rev	AGGGTCTTCTCCACAAAGTTACAATCACAATCTGAATTACTAAGAAG
NPR3-ΔANK-For	GAAATTGCTTGAAAGAATCGGTAAAGTTAATATATTAAGAAGACTGAC
NPR3-ΔANK-Rev	GTCAGTCTTCTTAATATATTAACTTTACCGATTCTTTCAAGCAATTTC
